# Helical allophycocyanin nanotubes absorb far-red light in a thermophilic cyanobacterium

**DOI:** 10.1126/sciadv.adg0251

**Published:** 2023-03-24

**Authors:** Christopher J. Gisriel, Eduard Elias, Gaozhong Shen, Nathan T. Soulier, David A. Flesher, M. R. Gunner, Gary W. Brudvig, Roberta Croce, Donald A. Bryant

**Affiliations:** ^1^ Department of Chemistry, Yale University, New Haven, CT 06520, USA.; ^2^ Department of Physics and Astronomy, and LaserLaB Amsterdam, Vrije Universiteit Amsterdam, De Boelelaan 1081, 1081 HV, Amsterdam, The Netherlands.; ^3^ Department of Biochemistry and Molecular Biology, The Pennsylvania State University, University Park, PA 16802, USA.; ^4^ Department of Molecular Biophysics and Biochemistry, Yale University, New Haven, CT 06520, USA.; ^5^ Department of Physics, City College of New York, New York, NY 10031, USA.

## Abstract

To compete in certain low-light environments, some cyanobacteria express a paralog of the light-harvesting phycobiliprotein, allophycocyanin (AP), that strongly absorbs far-red light (FRL). Using cryo–electron microscopy and time-resolved absorption spectroscopy, we reveal the structure-function relationship of this FRL-absorbing AP complex (FRL-AP) that is expressed during acclimation to low light and that likely associates with chlorophyll a–containing photosystem I. FRL-AP assembles as helical nanotubes rather than typical toroids due to alterations of the domain geometry within each subunit. Spectroscopic characterization suggests that FRL-AP nanotubes are somewhat inefficient antenna; however, the enhanced ability to harvest FRL when visible light is severely attenuated represents a beneficial trade-off. The results expand the known diversity of light-harvesting proteins in nature and exemplify how biological plasticity is achieved by balancing resource accessibility with efficiency.

## Graphical abstract

**Figure:**
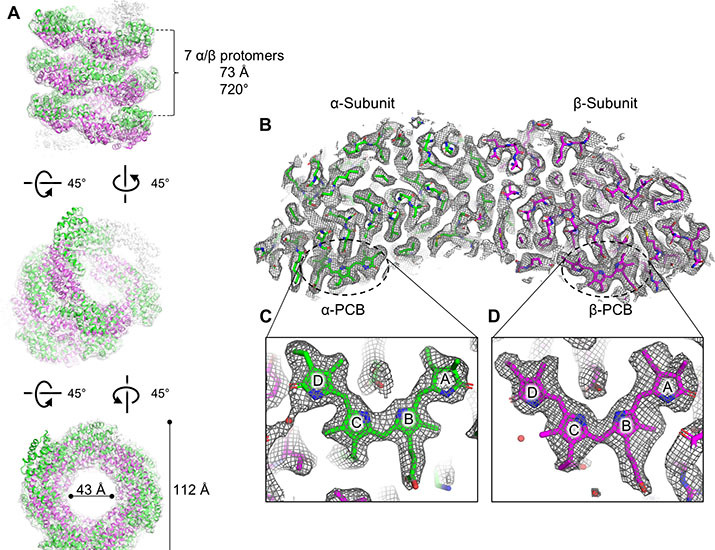

## Teaser

A far-red light-absorbing phycobiliprotein assembles as helical nanotubes to provide more photons for photosynthesis.

## INTRODUCTION

Photosynthetic reaction centers are photo-oxidoreductases that convert light energy into chemical potential energy (*1*). All reaction centers bind six key (bacterio)chlorophyll pigments that function in light harvesting, energy transfer, and electron transfer (*2*, *3*). However, because sunlight is a dilute source of energy, reaction centers alone would operate inefficiently. This is because the limited numbers and absorbance ranges of the (bacterio)chlorophyll molecules in their electron transport chains would limit the rate of photon capture and conversion (*4*, *5*). Therefore, all chlorophyll (Chl)–dependent phototrophs additionally bind either core antenna pigments or peripheral antenna complexes that associate with reaction centers to extend their absorbance cross sections. These antenna pigments substantially increase the numbers and wavelength ranges of photons that can be captured to drive photochemistry and greatly accelerate the rate of photon absorbance for photosynthesis. In contrast to the conserved reaction centers, phototrophs have collectively evolved a diverse array of peripheral light-harvesting complexes (*4*, *5*).

Cyanobacteria are thought to have first evolved the capacity for oxygenic photosynthesis, and today, they are responsible for about 10 to 15% of global carbon fixation. Like plants and eukaryotic green algae, cyanobacteria use two essential photo-oxidoreductases, photosystem I (PSI) and photosystem II (PSII), that work in series to oxidize water and produce strong reductants for carbon dioxide fixation (*6*). Because cyanobacterial PSI and PSII usually bind only Chl a and carotenoids, cyanobacteria must directly compete with plants, eukaryotic algae, and other phototrophs for light to support their metabolism. Most cyanobacteria produce water-soluble phycobiliproteins (PBPs) that strongly absorb light in the 550- to 650-nm range because of covalently bound, linear tetrapyrrole chromophores called bilins (*4*, *7*). Directed by assembly proteins, so-called linker proteins, PBPs assemble into supercomplexes known as phycobilisomes (PBSs), which associate with PSI and PSII to extend the wavelength range of light that can be used to drive photochemistry (*8*). The basic assembly unit is always a heterodimeric “protomer” containing one α-subunit and one β-subunit, each of which binds one or more bilins (*4*, *7*). Protomers oligomerize to form toroid-shaped (αβ)_3_ trimers and/or (αβ)_6_ hexamers, which can interact with linker proteins to form cylindrical stacks. The cylindrical stacks can organize forming a variety of PBS structures (*4*, *7*). The most common PBSs found in cyanobacteria have a hemidiscoidal morphology; three cylinders containing the PBP allophycocyanin (AP) associate with PSII at the thylakoid membrane surface and form a pyramidal core substructure, from which six peripheral rods composed of the PBP phycocyanin (and the PBP phycoerythrin, if present) radiate (*4*, *9*–*11*). Depending upon the organism and the environmental conditions, however, PBP complexes with different compositions and structures can assemble (*12*, *13*). Aside from the common hemidiscoidal architecture, sometimes only a single rod is formed (*4*, *14*). In other cases, cylinders containing only AP variants are formed that associate with the membrane without peripheral rods (*15*–*17*). Finally, several classes of PBS structure have been found and described (*4*). Nevertheless, despite the diversity in PBP arrangements that occur in nature, the structural building blocks of previously investigated PBPs are toroid-shaped trimers or hexamers that can form cylindrical stacks.

The competition for light and its optimal usage for photosynthesis led cyanobacteria to evolve numerous, facultative acclimation responses (*12*, *13*, *18*). One such response is low-light photoacclimation (LoLiP), which involves the expression of genes from an operon that encodes a far-red light (FRL)–absorbing AP; a putative Chl a–binding protein, IsiX, that is a member of the PsbC-IsiA superfamily of Chl a–binding proteins; and a cyanobacteriochrome photoreceptor (*18*). LoLiP is distinct from the recently characterized FRL photoacclimation (FaRLiP), in which the photosystems are altered to bind Chls d and f that also absorb FRL (*19*, *20*). FaRLiP also leads to the expression of several AP variants (ApcD2, ApcD3, ApcD5, ApcB2, and ApcE2) that form complexes that absorb FRL (*15*–*17*).

Despite binding the same phycocyanobilin (PCB) chromophore as conventional AP (λ_max_ = 650 nm) and the PBS terminal emitter, AP-B (λ_max_ = 670 nm) (*21*), the absorbance maximum of the FRL-absorbing AP expressed during LoLiP is bathochromically shifted by ~40 to 60 nm to ~710 nm (*17*, *18*, *21*). The FRL-AP expressed during LoLiP is composed of an α-subunit, ApcD4, and a β-subunit, ApcB3, each of which binds a single PCB. Although FRL-absorbing AP-family members can comprise multiple combinations of subunits (*17*), we will hereafter refer to ApcD4-ApcB3 as FRL-AP unless otherwise noted. PBSs produced during normal, high-light growth conditions are still produced during LoLiP, but the additionally produced FRL-AP in cells grown in low light does not copurify with PBS. Instead, FRL-AP is membrane-associated and copurifies after detergent solubilization with IsiX, which suggests the existence of a unique antenna complex specialized for harvesting FRL (*18*). The structural and functional bases of light absorbance by FRL-absorbing AP-family members are still unclear. To address these issues, we used single-particle cryo–electron microscopy (cryo-EM) and time-resolved spectroscopy to reveal the structure-function relationship of the FRL-AP (ApcD4-ApcB3) produced during LoLiP.

## RESULTS

### Characterization and cryo-EM

Soulier *et al.* (*17*, *18*) found that the *apcD4-apcB3* operon from the LoLiP strain *Synechococcus* sp. A1463 could be overexpressed in either *Escherichia coli* or *Synechococcus* sp. PCC 7002, but that expression in the cyanobacterium led to a better yield of fully chromophorylated FRL-AP in the soluble protein fraction of whole-cell extracts. FRL-AP was purified from cell extracts by immobilized metal affinity chromatography, and after concentration by centrifugal filtration, the protein had an intense steel-blue color (fig. S1A). SDS–polyacrylamide gel electrophoresis (SDS-PAGE) confirmed the presence of two subunits, His_10_-tagged ApcD4 (α-subunit) and ApcB3 (β-subunit), and Zn-enhanced fluorescence imaging confirmed that both were chromophorylated (fig. S1B). Some fluorescent dimeric forms were observed even after denaturation, which could represent disulfide cross-linked polypeptides produced under the oxidizing conditions in the gel (figs. S1, B and C). Unlike most other PBPs, FRL-AP contains several additional cysteine residues other than the cysteines to which PCB is covalently attached (*21*). Tryptic peptide mass fingerprinting verified that His_10_-tagged ApcD4 and ApcB3 were the major constituents (table S1) (*17*, *18*, *21*). Chymotryptic peptide mass fingerprinting established that the Asn residue at position 71 in ApcB3 was methylated, which is a characteristic of nearly all PBP β-subunits and is required for efficient energy transfer (*22*). This methylation is not present when the protein is produced in *E. coli*. The absorbance spectrum of the purified protein showed a very broad absorbance band centered at 621 nm and a very sharp and more intense absorbance band at 709 nm (fig. S1D). The FRL-AP exhibited a very small Stokes shift with a 77 K fluorescence emission maximum at 714 nm (fig. S1E). Size exclusion chromatography indicated that FRL-AP occurred as a broad distribution of oligomers (fig. S2). Only the main peak from the size exclusion chromatography was used for single-particle cryo-EM to minimize heterogeneity.

**Fig. 1.**
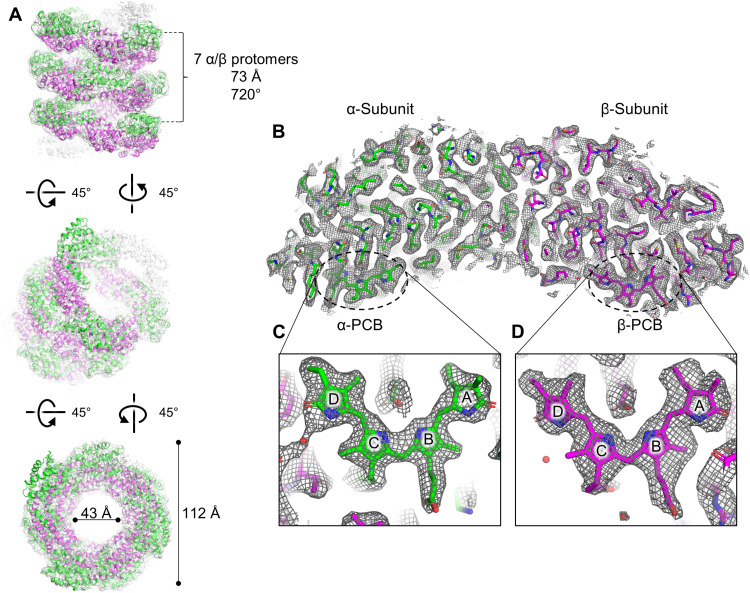
Overview of FRL-AP helical nanotube structure. (**A**) Three views of the unsharpened FRL-AP map. Nine protomers are shown in which α-subunits are colored green and β-subunits are colored magenta. In the top panel, the helical parameters are labeled, and in the bottom panel, the outer and inner diameters of the helical nanotube are labeled. Note that the number of subunits shown is arbitrary because the data are processed with helical parameters. (**B**) Cross section of a single protomer at 5σ. (**C**) α-PCB within the cryo-EM map. (**D**) β-PCB within the cryo-EM map. In (B) and (C), the map is shown at 7.5σ and the pyrrole rings of the PCBs are labeled.

Single-particle cryo-EM data for FRL-AP were collected, and individual particles were observed in the micrographs (fig. S3). The single-particle reconstruction exhibited a helical configuration with a global resolution of 2.89 Å (see Materials and Methods and fig. S4) with local resolution ranging from 2.5 to 3.3 Å (fig. S4). FRL-AP protomers were modeled into the map to create the helical FRL-AP structure (Fig. 1). Each (αβ) protomer binds two PCBs (one per subunit), one anion tentatively modeled as Cl^−^ and 110 water molecules.

### Structural analysis

**Fig. 2.**
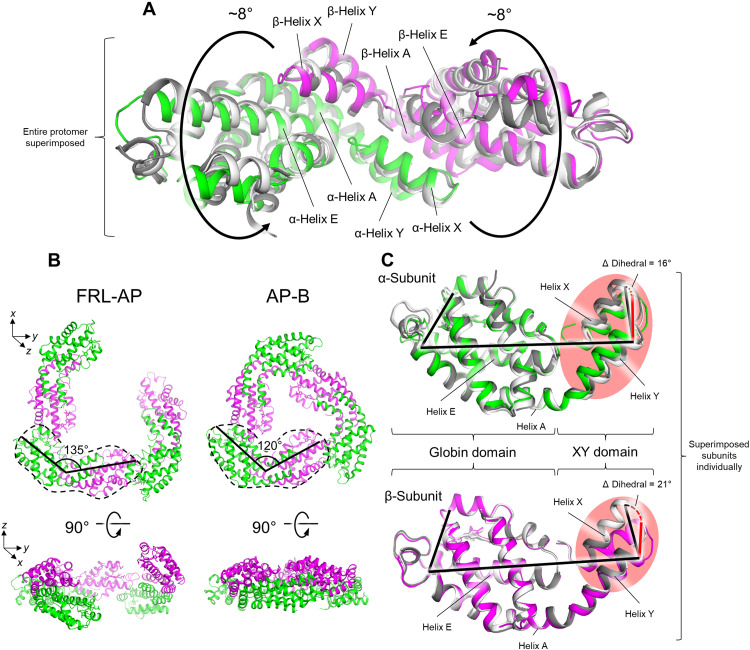
Basis for helix formation of FRL-AP. (**A**) A protomer of FRL-AP is shown, superimposed with the structures of AP [white; Protein Data Bank (PDB): 4RMP (*41*)] and AP-B [gray; PDB: 4PO5 (*24*)]. The twist between the two subunits unique to this FRL-AP is denoted with the rotating arrows. (**B**) Comparison of three protomers in the FRL-AP helical structure to the trimeric toroid of AP-B. Two views of each are shown. In the top view, a single protomer from each is circled in a dashed line and the approximate angles between the α- and β-subunits in the *xy* plane are labeled. Although AP is not included, its structure at this level of detail is essentially identical to that of AP-B. (**C**) Superimposed individual subunits of FRL-AP (colored) with AP (white) and AP-B (gray). Regions of the XY domain that poorly superimpose are shown with a red background. Dihedral angle measurements are additionally shown, which allow for visualization of the unique orientation between the globin and XY domains of FRL-AP relative to AP and AP-B.

Each repeating helical unit of FRL-AP comprises seven protomers that span 720° over 72.6 Å (Fig. 1A). Relative to the protomer structures of other AP-family representatives, the α- and β-subunits are slightly twisted in opposite directions, each ~8°, resulting in a more open configuration that facilitates the helical assembly (Fig. 2A and fig. S5). Consequently, the α- and β-subunits of FRL-AP form an angle of ~135° in the *xy* plane, whereas the corresponding angle in the structures of other AP-family representatives is ~120°, and there is a slight separation along the *z* axis, ~10° (Fig. 2B, bottom left).

**Fig. 3.**
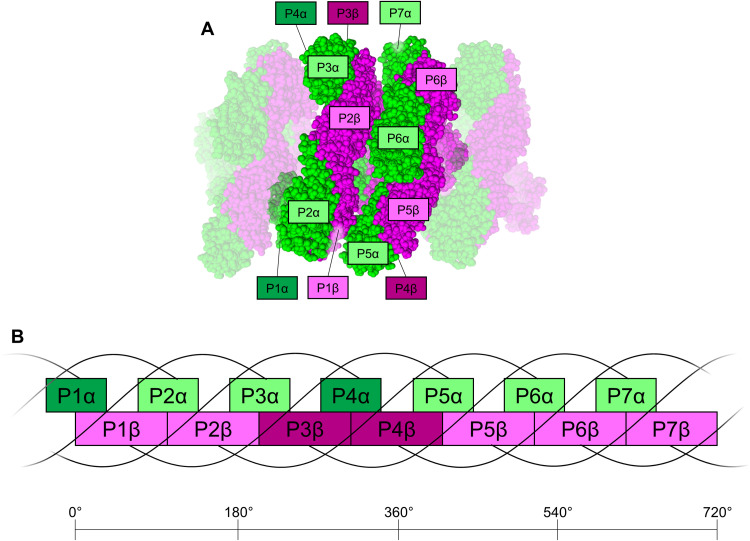
Subunit interactions in the helical FRL-AP nanotube. (**A**) Protomers in the helical nanotube are shown using a sphere representation in which α-subunits are colored in green and β-subunits are colored in magenta. Seven central protomers are shown, which composes a single repeating helical unit (720°), and further protomers are shown extending the helical nanotube for reference. In the repeating helical unit, the α- and β-subunits are labeled P1 to P7. Those with dark labels are in the back of the view shown and therefore cannot be seen in this view. (**B**) 2D schematic representation of (A). Each α-subunit interacts with three β-subunits: that of its own protomer, the β-subunit from the protomer before it, and the one from the protomer below it (four protomers prior in the helix). Each β-subunit interacts with two other β-subunits, from the protomers before and after it, and three α-subunits, from its own protomer, from the next protomer, and from the protomer above it (four protomers later in the helix).

In the helical configuration, an α-subunit does not interact with any other α-subunit, but it interacts with three β-subunits: that of its own protomer, that of the prior protomer in the helix, and that of the protomer in the helical level below it (Fig. 3). Hereafter, we use the nomenclature of PBP secondary structure first introduced in (*23*) (fig. S6). From the perspective of quaternary structure analysis, many protein-protein contacts are similar to those found in conventional toroidal PBP complexes. Only the interaction between helix H of the α-subunit and helix H of the β-subunit from the protomer in the helical level below it is unique to the helical configuration. While we can resolve the residues at this interface (fig. S7), it is unclear which, if any, are involved in stabilizing this interaction. It seems unlikely that unique residues within this interface are involved in helix stabilization because none of the residues at this interface are conserved among ApcD4 or ApcB3 sequences (fig. S8). In addition to this contact with the α-subunit above it, a β-subunit interacts with two other β-subunits, those from the protomers before and after it in the helix, and extensively with the α-subunit of its own protomer. These interactions are also observed in AP, AP-B, and other PBPs.

In all PBP protomers, the XY domain of one subunit interacts primarily with helices E and A of the globin domain of the other subunit (Fig. 2A). Superimposing the individual α- and β-subunits of FRL-AP onto the corresponding subunits from other AP-family representatives (i.e., AP and AP-B) reveals that the XY domain of each subunit is positioned differently in FRL-AP (Fig. 2C), which is the cause of the more open configuration that results in helix formation. In the α-subunit, the entire XY domain is rotated ~16°, relative to the globin domain. In the β-subunit, helix X and the XY loop are substantially shifted, ~21°.

To gain further insight into the structural basis of helix formation, we constructed a sequence alignment of various AP subunits (fig. S8) and identified residues specific to ApcD4 and ApcB3, the subunits in a FRL-AP protomer, that correlate to the structural shifts in their XY helical domains relative to the structures of AP-B and AP (Fig. 2) and that are implicated in helix formation. In ApcD4, the α-subunit of FRL-AP, position 11 is Ser instead of Ala that is found in all other α-subunit sequences (fig. S8A). In the FRL-AP structure, this α-Ser^11^ donates an H bond to the side chain of α-Glu^21^ that is conserved among all the α-subunit sequences (fig. S8A). In the structures of AP and AP-B, which do not have this α-Ser^11^ interaction, α-Glu^21^ accepts an H bond from the backbone amide nitrogen atom of the residue at position 19, which is within the XY loop, likely stabilizing the position of the loop. In the FRL-AP structure, α-Glu^21^ is not within H-bonding distance of the XY loop, and the loop is moved away, probably due to α-Ser^11^ occupying α-Glu^21^ by H bonding (fig. S9A). In addition, the backbone amide nitrogen atom of α-Glu^21^ donates an H bond to α-Ser^19^ in the AP-B and AP structures, but because of the destabilized loop, α-Ser^19^ in the FRL-AP structure is moved out of H-bonding distance, causing helix Y to be one residue shorter (and the XY loop to be one residue larger) (fig. S9A). To summarize, the H bond donated from α-Ser^11^ in ApcD4 results in destabilization of the XY loop and shifting of helix Y (Fig. 2C). This is likely to contribute to the overall twist of the protomers in the FRL-AP structure (Fig. 2A).

In the β-subunit sequence comparison, position 4 is Ala in all β-subunit sequences except that from FRL-AP, ApcB3, in which it is instead Thr (fig. S8B). The hydroxyl moiety of the β-Thr^4^ side chain in the FRL-AP structure donates an H bond to the backbone carbonyl oxygen atom of β-Ala^98^ (fig. S9B). In the structures of AP and AP-B, this H bond is also present but is instead donated from the backbone amide nitrogen atom of position 4, which is Ala. Because of this difference in H bonding, helix X begins in a slightly different position, which propagates through the length of the helix, resulting in a major offset relative to helix X in the structures of AP and AP-B (Fig. 2C). An additional difference in helix X of the FRL-AP structure is found at position 11, which is Pro, whereas it is instead Thr, Ser, or Ala in other β-subunit sequences (fig. S8B). β-Pro^11^ results in a bend in helix X of the β-subunit (fig. S9B), which also contributes to the difference in structures (Fig. 2C). This also alters the position of the XY loop that causes different interactions with the α-subunit. For example, β-Tyr^18^ is conserved in all the sequences and, based on our structural analysis, is inserted between helices A and E of the α-subunit of the same protomer (fig. S9C). In the structures of AP and AP-B, the hydroxyl moiety of β-Tyr^18^ donates an H bond to carboxylate side chains in the α-subunit and is inserted at an angle of ~40° relative to helix E of the α-subunit. In the FRL-AP structure, α-Tyr^18^ does not exhibit H-bonding interactions and is inserted at an angle of ~60°.

**Fig. 4.**
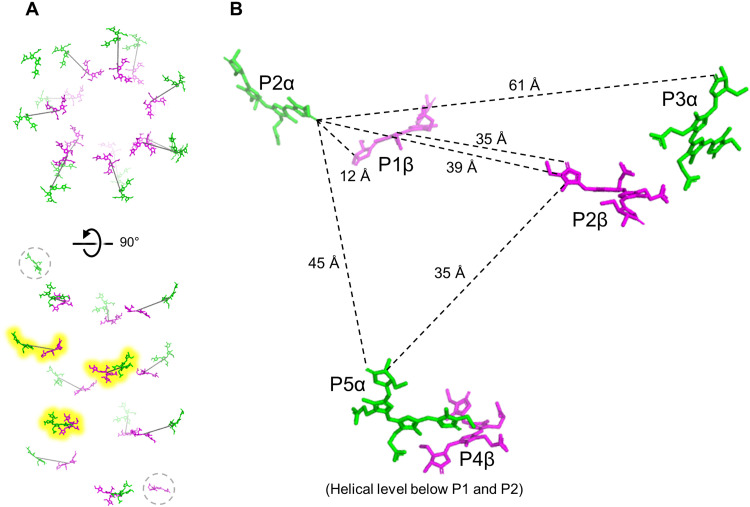
PCB chromophore organization in FRL-AP helical nanotubes. (**A**) Overview of the arrangement of PCB chromophores in the FRL-AP helical nanotube structure. The PCBs of 13 protomers are shown. Lines are drawn connecting the centers of a pair of PCBs for reference (one from each subunit of adjacent protomers). The three pairs that are highlighted in yellow are shown in a closer view in (**B**). Disconnected terminal PCBs are circled with a dashed gray line. (B) Distances (edge to edge) of PCBs in the FRL-AP helical structure. The uppermost β-PCB is labeled as being bound in the first protomer (P1β), although this number is arbitrary and serves as a reference for denoting the protomers that bind the other PCBs shown.

The arrangement of PCB cofactors in the FRL-AP nanotubes is shown in Fig. 4. β-PCBs line the inside of the FRL-AP helix, and α-PCBs form an outer layer, which is similar to the arrangement observed in toroidal trimeric PBPs. The closest edge-to-edge distance, ~12 Å, for PCBs in the helical FRL-AP complex occurs between an α-PCB and the β-PCB of the nearest adjacent protomer. This is essentially identical to the corresponding distances in other PBPs. Each nanotube must begin/end with a relatively isolated protomer, i.e., one helical end contains either an α-PCB or a β-PCB, each ~35 Å from any other PCB (Fig. 4A).

**Fig. 5.**
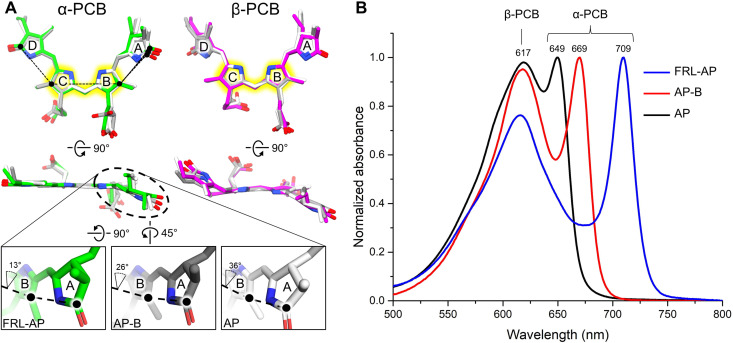
Comparison of PCB structure and absorbance spectra for FRL-AP, AP-B, and AP. (**A**) Superimposed α-PCB (left) and β-PCB (right) from FRL-AP (α-PCB is colored green and β-PCB is colored magenta), AP-B (gray), and AP (white). PCBs are superimposed by the atoms highlighted in yellow. The black dashed lines in the top row designate which atoms were used to calculate the torsion angle. The bottom panels show magnified views of the A and B rings in the α-PCB for each, and the corresponding dihedral angle is labeled. (**B**) Absorbance spectra of FRL-AP (helical ApcD4-ApcB3 from *Synechococcus* A1463), the PBS terminal emitter AP-B (trimeric ApcD1-ApcB1 from *Synechococcus* A1463), and conventional AP (trimeric ApcA1-ApcB1 from *Leptolyngbya* sp. JSC-1). The primary PCB contributor to each of the two peaks of each absorbance spectrum is labeled; however, it should be noted that the peaks labeled “α-PCB” all exhibit an equivalent broadening and blue shift of ~30 nm when not complexed with β-subunits (*17*, *18*, *21*). The spectra of the individual isolated subunits are shown in fig. S10.

Like all other AP-family members, FRL-AP binds PCB chromophores whose absorbance properties are determined by their protein environment. Peng *et al*. (*24*) observed a correlation between increased planarity of the PCB rings and bathochromic shifting of the absorbance maximum, and Soulier and Bryant (*21*) recently suggested that the α-PCB of FRL-AP exhibits the greatest planarity among PBPs binding PCB. To investigate the correlation of PCB structure to absorbance, we superimposed rings B and C, which are essentially coplanar, of the α- and β-PCBs from FRL-AP, AP-B, and AP (Fig. 5A). For all three, the pyrrole rings of β-PCBs are less coplanar than the α-PCBs, which is consistent with proposals that the β-PCB binding site of FRL-AP is less constrained than that of the α-PCB and with its broader contribution to the absorbance spectra of protomers (*17*, *21*, *24*). The α-PCBs exhibit more distinct differences: Whereas rings B, C, and D are nearly coplanar, ring A is shifted out of plane in each, but least so for FRL-AP. The specific dihedral angles among the α-PCB rings are shown in Fig. 5A. The increasing degree (FRL-AP ➔ AP-B ➔ AP) to which ring A is coplanar with the rest of the α-PCB corresponds nicely to the 680-, 650-, and 625-nm absorbance maxima of isolated α-subunits (fig. S10) and to the 709-, 669-, and 649-nm far-red absorbance maxima of their oligomeric states (Fig. 5B).

### Energy transfer in FRL-AP nanotubes

**Fig. 6.**
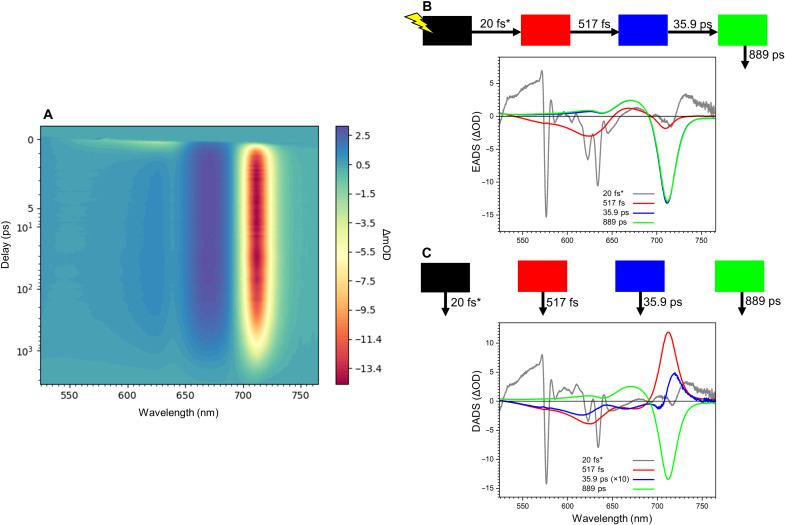
Time-resolved absorption spectroscopy of FRL-AP. (**A**) Absorption change profile of FRL-AP nanotubes excited at 580 nm. (**B**) Evolution-associated difference spectra (EADS) fit from (A) and the associated model scheme. (**C**) Decay-associated difference spectra (DADS) fit from (A) and the associated model scheme. The third (blue) DADS has a small amplitude and is therefore multiplied 10 times for clarity. In (B) and (C), asterisks indicate that the rate was fixed in the global analysis.

To investigate the energy transfer dynamics of the FRL-AP nanotubes, we performed transient absorption spectroscopy, selectively exciting the β-PCBs at 580 nm and monitoring the change in optical density (ΔOD) from 500 to 780 nm (Fig. 6A). The negative signal in the 550- to 650-nm region corresponds to the ground-state bleach and stimulated emission of excited β-PCBs (*21*), which decays on a sub-picosecond time scale. Concomitantly, the negative signal centered at ~709 nm rises, corresponding to an increase in the excited-state population of the α-PCBs (*21*). The subsequent dynamics are dominated by the decay of excited α-PCBs to their ground-state roughly on a nanosecond time scale.

To obtain a quantitative description of the data, we performed a global analysis (Fig. 6, B and C). The initial evolution-associated difference spectrum (EADS) represents the spectrum at time zero (Fig. 6B, gray line), and it contains contributions from coherent artifacts and probably excited vibronic modes; however, it shows some negative amplitude centered at 710 nm arising from a small population of directly excited α-PCBs. The initial EADS (gray) evolves in 20 fs to the second EADS (red), which shows mainly the ground-state bleach/stimulated emission of the β-PCBs (525 to 655 nm) and a small portion of ground-state bleach/stimulated emission from the α-PCBs (690 to 730 nm). The ground-state bleach/stimulated emission in the α-PCB spectral region is similar in the first (gray) and second (red) EADS, indicating that there is no ultrafast 20-fs excited-state energy transfer (EET) to the α-PCBs. The second (red) EADS evolves to the third (blue) EADS with a time constant of 517 fs, which represents the main energy transfer time from β-PCBs to α-PCBs. This is clearly visible in the second decay-associated difference spectra (DADS) in Fig. 6C, which shows the typical EET shape with negative features at 690 to 740 nm and positive features in the 525- to 690-nm region. The third (blue) DADS has a time constant of 35.9 ps and a very small amplitude. It also has an energy transfer component from β-PCBs and “blue” α-PCBs (negative feature near 705 nm) to redder α-PCBs. We suggest that this DADS arises from the more “isolated” α-PCB and β-PCB chromophores that are found on the helical termini. The amplitude is approximately proportional to what would be expected for the two terminal PCBs on a nanotube comprising ~13 protomers (Fig. 4). The fourth (green) DADS with a time constant of 889 ps represents the decay to the ground state of the excited α-PCBs. To confirm the short excited-state lifetime of FRL-AP nanotubes, we also performed time-correlated single-photon counting on the FRL-AP sample, exciting at 470 nm and monitoring the α-PCB emission at 710 nm (see Materials and Methods). The decay was satisfactorily fitted with one component of 825 ps, which is similar to the excited state lifetime determined by the time-resolved absorption spectroscopy (889 ps). Collectively, these data indicate that the main EET dynamics of FRL-AP helical nanotubes arise from rapid EET within the α-PCB/β-PCB pairs on a sub-picosecond time scale.

To obtain a more detailed understanding of EET in FRL-AP and to connect the measurements with the structural data, we modeled the EET dynamics using Förster resonance energy transfer (FRET) theory. The description of how parameters were retrieved for calculating FRET rates can be found in text S1 and figs. S11 and S12. Simulated EET dynamics neatly capture the dynamics that are observed in the experiment, in which most of the energy initially residing on the β-PCBs is transferred to the α-PCBs within ~500 fs (fig. S13A). It can moreover be observed that a residual part of the energy on β-PCBs is transferred at a slower rate to the α-PCBs, which is due to EET from the isolated terminal PCB. The temporal excited-state population of the β-PCBs was fitted to a tri-exponential function in which two components represent the EET processes and the third component represents the excited-state decay (fixed lifetime component at 889 ps; fig. S13B). The time constants from the fitting are 488 fs and 39.7 ps with an amplitude ratio of 12:1, which agree very well with the experimental global analysis results (Fig. 6C).

## DISCUSSION

**Fig. 7.**
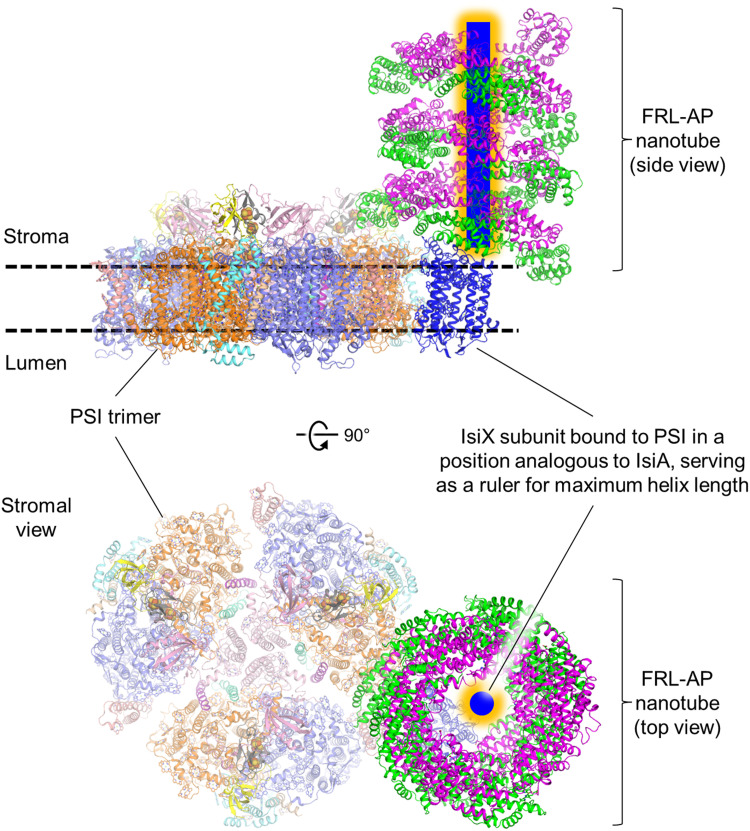
Proposed interaction of FRL-AP nanotubes, IsiX, and PSI. Two views are shown in which the helical FRL-AP nanotube is positioned perpendicular to the membrane. The C-terminal extension of IsiX (blue with orange glow) is proposed to occupy the space in the center of the FRL-AP nanotube, and based on its strong similarity to IsiA (fig. S14), the transmembrane region of IsiX (blue) is proposed to bind to the periphery of PSI. The C-terminal extension could serve as a molecular ruler to limit the length of the helix and establish the optimal nanotube length for energy transfer to IsiX. Note that although we have shown the orientation of the FRL-AP nanotube perpendicular to the membrane, which is analogous to the configuration of CpcL-PBS (*14*), it remains possible that the nanotubes could lie parallel to the membrane plane similar to core cylinders in hemidiscoidal PBS. Further work is needed to ascertain the orientation in vivo.

It is important to contextualize the spectroscopic data with how PBPs might assemble with a photosystem in vivo. Insight is obtained by considering that FRL-AP is coproduced with and copurifies with IsiX (*18*), which shares homology with IsiA. IsiA is up-regulated under conditions of iron starvation in cyanobacteria and associates with PSI in a ring-like structure (*25*). IsiX, which is regulated instead by light availability, shares ~56% sequence identity to IsiA and is predicted to have the same tertiary structure and Chl-coordinating residues as IsiA (fig. S14). The major difference between IsiX and IsiA is that IsiX contains a C-terminal extension of 127 residues. On the basis of these observations, it seems reasonable to suggest that FRL-AP, IsiX, and PSI assemble into a supercomplex. Thus, we propose the model shown in Fig. 7, which suggests that the C terminus of IsiX serves as a ruler, threading through the center of a FRL-AP nanotube, potentially limiting and optimizing its length and bringing it into proximity of PSI for energy delivery. This model is consistent with the fact that FRL-AP (ApcD4-ApcB3) is not found in the soluble PBP-containing fraction and instead associates with an insoluble, membrane-containing fraction together with IsiX (*18*). Furthermore, this arrangement would be analogous to the CpcL-PBS found in some *Anabaena* species, which exhibits single phycocyanin cylinders/rods associated with PSI (*14*). Alternate ways of IsiX associating with the FRL-AP nanotubes cannot be ruled out, such as the wrapping of the IsiX C-terminal domain around the outside of the nanotube. In any case, energy transfer from FRL-AP to Chl a in PSII with typical site energy (Q_y_ = ~680 nm) would be quite inefficient (fig. S15); thus, it seems feasible that FRL-AP nanotubes associate with PSI, which has red Chl forms (*26*) as we propose (Fig. 7). This proposal also fits nicely with the fact that typical PBS complexes are not altered during LoLiP, so conventional hemidiscoidal PBS could still associate with PSII.

A PCB excited by an incident photon will result in EET along the FRL-AP nanotube, so it is also important to consider the length of a FRL-AP nanotube. In our preparation, the nanotube length is heterogeneous. Most individual particle images appear to be ~10 to 13 protomers, which is also observed in two-dimensional (2D) classification (fig. S3) and is consistent with the peak in the size exclusion chromatogram; however, the particle selection approach also targets particles of ~10 to 13 protomers, and smaller helices become more challenging to identify in the micrographs. In vivo, we can imagine that the length of FRL-AP nanotubes could be limited by the C-terminal extension of IsiX that serves as a molecular ruler as described above, which could decrease our estimate of 10 to 13 protomers per nanotube. On the other hand, an AP cylinder in hemidiscoidal PBS contains 12 protomers, which is similar to the ~10 to 13 protomers that we observe as the major population in the cryo-EM data. Using a model of 13 protomers, when the excitation is in the most peripheral chromophore relative to IsiX and PSI, our FRET model allows us to calculate that energy would migrate to the other end of the nanotube in ~600 to 800 ps (text S2 and fig. S13C). The excitation energy transferred to an irreversible trap located at one end of a 13-protomer nanotube (containing 26 PCBs) would be below 50%, but the efficiency increases to ~65% using a model with 13 PCBs (text S3 and fig. S16). More investigations are needed to better understand the nanotube length of FRL-AP in vivo, the spectroscopic properties of the Chls of IsiX, and the way in which FRL-AP nanotubes specifically associate with PSI for light excitation energy transfer.

Despite the possible low efficiency of EET, FRL-AP nanotubes are synthesized under extremely stressful conditions, and the benefit to the organism of harvesting additional far-red photons may outweigh the suboptimal quantum efficiency of this light-harvesting system when visible light is severely limiting. This is reminiscent of the FaRLiP response of several cyanobacteria, for which it has been shown that the increase in harvestable photons through the accumulation of the red-shifted Chl f and/or Chl d molecules in the photosystems outweighs the losses arising from the reduced quantum efficiencies (*27*). The production of FRL-AP nanotubes by thermophilic *Synechococcus* sp. is therefore an interesting strategy that has evolved to make additional light accessible for driving photosynthesis when visible light is limited.

## MATERIALS AND METHODS

### Strains and growth conditions

Wild-type *Synechococcus* sp. PCC 7002 (hereafter *Synechococcus* 7002) and related strains were grown at 37°C in medium A^+^ (medium A plus sodium nitrate) as previously described (*18*). *Synechococcus* A1463, also known as *Synechococcus* sp. 63AY4M1 (National Center for Biotechnology Information Taxonomy ID: 1353263), is a uni-cyanobacterial enrichment culture of a low light–adapted *Synechococcus* sp. ecotype isolated from Mushroom Spring, Yellowstone National Park (*18*). A draft version of the genome sequence of *Synechococcus* A1463 is available in GenBank as accession GCA_002760395.1. The *apcD4* and *apcB3* genes of *Synechococcus* A1463 were amplified by polymerase chain reaction (PCR) and cloned into expression vector pAQ1Ex, producing plasmid pAQ1Ex-*apcD4apcB3* as previously described (*18*, *21*). The resulting plasmid was transformed into *Synechococcus* 7002, which was verified through colony PCR analysis, to produce strain *Synechococcus* 7002*-apcD4B3*.

### Purification of recombinant ApcD4-ApcB3

Recombinant FRL-AP (ApcD4-ApcB3) was overproduced and purified from *E. coli* as previously described (*17*, *21*). Recombinant FRL-AP (ApcD4-Apc3) was also overproduced in *Synechococcus* 7002*-apcD4B3* cells and purified via an N-terminal [His]_10_-tag on the ApcD4 subunit. Liquid cultures of *Synechococcus* 7002*-apcD4B3* were grown in A^+^ growth medium amended by addition of spectinomycin (100 μg ml^– 1^). Overproduction of ApcD4-ApcB3 FRL-AP was monitored by measuring the far-red absorbance band at 709 nm. Cells were harvested by centrifugation at 6900*g* and resuspended in isolation buffer [100 mM K phosphate (pH 7.0) and 50 mM NaCl]. A cell lysate was produced by passing cell suspensions three times through a chilled French pressure cell at 124 MPa. After removal of unbroken cells and cell debris by centrifugation at 6900*g* for 10 min, the lysate was further clarified by centrifugation at 12,600*g* for 30 min to pellet membranes. Before immobilized metal affinity chromatography as previously described (*17*, *21*), 1 mM l-histidine and 0.02% *n*-dodecyl-β-d-maltoside (β-DM) were added to the soluble fraction. The resulting solution was loaded onto a column packed with high-density Ni–nitrilotriacetic acid resin. The column was initially washed with two column volumes of washing buffer A [100 mM K phosphate (pH 7.0), 50 mM NaCl, 2 mM l-histidine, and 0.02% β-DM] and three column volumes of washing buffer B [100 mM K phosphate (pH 7.0), 5 mM NaCl, and 2 mM l-histidine]. The [His]_10_-ApcD4ApcB3 protein complexes were subsequently eluted with the elution buffer [100 mM K phosphate (pH 7.0), 5 mM NaCl, and 50 mM l-histidine]. The purified protein was concentrated to approximately 10 mg ml^−1^ using Millipore centrifugal concentrators (50-kDa cutoff; EMD Millipore, Darmstadt, Germany).

### Analytical characterization

A Cary 14 spectrophotometer modified for online data acquisition (On-line Systems Inc., Bogart, GA) was used to measure absorbance spectra of the FRL-AP (fig. S1D). Steady-state fluorescence emission spectra were measured at 77 K using an SLM 8000C spectrofluorometer modified for digital data acquisition by On-line Systems Inc. (Bogart, GA) (fig. S1E). The purity of the isolated protein was verified by PAGE in the presence of sodium dodecyl sulfate as described previously (fig. S1C) (*17*, *21*). The presence of PCB was detected by Zn-enhanced fluorescence (fig. S1B) (*16*, *19*). Protein composition was evaluated by tryptic and chymotryptic mass (MS-MS) fingerprinting spectrometry as described previously (table S1) (*16*, *18*, *19*). To perform size exclusion chromatography, the FRL-AP sample was loaded onto a HiLoad Superdex 200 16/600 column equilibrated with a buffer containing 50 mM K phosphate (pH 7.0) and 10 mM ethylenediaminetetraacetic acid. The major peak fraction (~4 ml) was collected and concentrated for use in cryo-EM (fig. S2).

### Cryo-EM grid preparation

The FRL-AP (ApcD4-ApcB3) protein solution isolated from *Synechococcus* 7002 was plunge-frozen using a Thermo Fisher Scientific Vitrobot. A 3-μl aliquot of the protein solution at 1.4 mg of protein per milliliter was applied to a glow-discharged (30 s, 25 mA) holey-carbon Quantifoil 2/1 Cu 300-mesh electron microscopy grid (Electron Microscopy Sciences). The grid was blotted for 3 s, plunged into liquid ethane, and stored in liquid nitrogen until data collection. The Vitrobot system temperature was 4°C and was set to 100% humidity.

### Cryo-EM data collection

Cryo-EM data were collected with a Titan Krios G2 transmission electron microscope (Thermo Fisher Scientific/FEI) operated at 300 kV with a Gatan K3 direct electron detector. The defocus range was set to −0.8 to −2.0 μm, and the nominal magnification was ×105,000. The super-resolution pixel size was 0.413 Å, and the GIF setting was a slit size of 20 eV. The dose rate was 23.6 e^−^ physical pixel^−1^ s^−1^ with 1.76 s per exposure comprising 44 images in a stack. Thus, the total dose was 59.8 e^−^ (Å) ^−2^. SerialEM was used to collect 10,755 micrograph movies.

### Cryo-EM data processing

Cryo-EM data processing was performed with RELION 3.1 (*28*). The processing workflow is shown in fig. S3. Initial motion correction, alignment, and dose weighting were performed with MotionCor2 (*29*), and the contrast transfer functions were estimated with Ctffind-4.1.13 (*30*). An initial set of ~750 particles, whose 2D classes appeared to support a helical configuration (fig. S3), were selected manually. These 2D classes were used as templates for Autopicking of the entire dataset, which yielded 601,552 particle selections. Subsequent 2D classification of this larger particle set was allowed for the removal of some classes leading to a particle set containing 509,505 particles. The InitialModel function of RELION 3.1 was used to create an ab initio map reconstruction that appeared helical. Using this as an input 3D map, 3D classification was used to isolate one class that clearly reconstructed to the highest resolution, and this was subsequently refined to a map at 4.22 Å. Rounds of contrast transfer function refinement and Bayesian polishing led to a nonsymmetrized map at 3.33 Å showing clear helical symmetry. Particle coordinates were used for re-extracted and refined using helical parameters, producing a reconstruction with helical symmetry at 3.72-Å resolution. Rounds of Bayesian polishing and contrast-transfer function refinement resulted in a 3D reconstruction at 2.93-Å resolution. A helical model containing 12 protomers was constructed using Coot (*31*), and the molmap function in UCSF Chimera (*32*) was used to create a 15-Å map of this structure. This was used to mask signal from the previous refinement job, outside of which the signal was subtracted in RELION 3.1 for focused refinement of the remaining signal (i.e., the signal corresponding to the protomers). A final round of refinement, sharpening, and masking led to the final map at 2.89-Å resolution.

### Model building

For model building, an initial model of FRL-AP was generated by creating homology models of ApcD4 and ApcB3 from the α- and β-subunits isolated from the crystal structure of AP-B from *Synechocystis* sp. PCC 6803 [Protein Data Bank (PDB): 4PO5] using SwissModel (*33*). These were fit into the cryo-EM map using UCSF Chimera (*32*), and the structures were edited manually using Coot (*31*). Subsequently, Phenix real_space_refine (*34*, *35*) was used for automated model refinement. Model statistics can be found in table S2. Some weak signal is found in the center of the nanotube that could not be interpreted and may correspond to disordered [His]_10_-tags of ApcD4 subunits.

### Transient absorption

For transient absorption experiments, the laser system consisted of a mode-locked Ti:Sa oscillator (Mira 900, Coherent Laser Group, Santa Clara, CA), in combination with a regenerative amplifier (RegA 9050, Coherent Laser Group, Santa Clara, CA). This provides ~70-fs pulses at a repetition rate of 40 kHz with a center wavelength of 800 nm. The pulses were then split in two with a beam splitter in a 20:80 intensity ratio. The 80% intensity pulses were directed through an optical parametric amplifier (OPA 9400, Coherent Laser Group, Santa Clara, CA) and tuned to 580 nm to provide the pump pulses. A band-pass filter was used to restrict the full width at half maximum of the pulses to 10 nm. The 20% pulses were focused in a sapphire crystal to create the white-light continuum for the probe pulses. The pump and probe pulses were intermittently turned on and off on a shot-to-shot basis by means of two acousto-optic modulators (S/N:1002 – O191168 MTS40-A3-750.850, AA Opto-Electronic, Orsay, France) that were controlled by a delay generator (DG645, Stanford Research Systems, Sunnyvale, CA), which was synced to the regenerative amplifier frequency. This was allowed for active dark current and scatter correction. The probe pulses were dispersed using a spectrograph (250IS, Chromex Inc., Albuquerque, NM) and then detected on a 1024-pixel charge-coupled device camera (FL3030, Entwicklungsbuero EB Stresing, Berlin, Germany), covering ~240 nm. The delay between the pump and the probe pulses was created by moving a retroreflector mounted on a delay stage. In this way, a delay of up to 3.5 ns could be measured. The polarization between the pump and the probe pulse was set at magic angle (54.7^o^) by means of a Berek’s variable waveplate. The sample was measured in a 1-mm quartz cuvette, which was constantly shaken. A power study was performed to exclude the presence of annihilation processes. The excitation pulse energy was set to 7.5 nJ for the measurement.

### Global analysis

The global analysis of the transient absorption experiment was performed using the Glotaran 1.5.1 software (*36*), yielding consecutively the DADS and EADS. In the global analysis in the kinetic model that yields the DADS, the components decay in parallel with their associated kinetic rates. In the kinetic model that yields the EADS, they instead evolve according to a unidirectional sequential scheme (EADS_1_➔EADS_2_➔EADS_n_). The instrument response function was modeled as a Gaussian function with a full width at half maximum of 96 fs.

### Time-correlated single-photon counting

The time-correlated single-photon counting measurements were performed using FluoTime 200 (PicoQuant, Berlin, Germany). The measurements were performed at 10°C, and the sample concentration was less than 0.05 cm^−1^ to avoid reabsorption. The excitation was provided at 470 nm at a repetition rate of 10 MHz at an average power of 162 μW. A power study was performed to ensure the absence of any power-dependent effects in the measurement. The fluorescence was detected at 710 nm. The sample was constantly stirred. The instrument response function was determined by measuring the decay of pinacyanol iodide dye in methanol (6-ps lifetime). The decay traces were fitted using the TRFA Data Processor Advanced Software (*37*).

### Steady-state spectroscopy

The absorption spectrum of FRL-AP used for FRET calculations was measured using a Varian Cary 4000 UV-Vis spectrophotometer at room temperature. The emission spectra were measured at room temperature with a HORIBA Jobin-Yvon Fluorolog-3 fluorometer at an OD < 0.05 cm^−1^.

### Calculation of parameters for modeling FRET in FRL-AP

The coordinates of the bilins were extracted from the FRL-AP cryo-EM structure. Missing hydrogen atoms were added using UCSF Chimera (*32*). Then, a geometry optimization protocol was performed using a B3LYP-D3BJ/TZ2P level of theory restraining the positions of all atoms except hydrogen. Subsequently, the first 20 excited states were calculated for the optimized geometry using the time-dependent density functional theory (*38*) B3LYP-D3BJ/TZ2P level of theory using the Davidson approach implemented in the computer program ADF2021.101 (*39*). As an internal check, the methodology used was tested on the PEB α19 chromophore from the PE545 system (PDB: 1XG0) (*40*), and the orientation of the S_1_ transition dipole moment found was very similar to previously calculated results (*40*).

## Supplementary Materials

### This PDF file includes:

Supplementary Texts S1 and S2 Figs. S1 to S16 Tables S1 and S2 References

## Notes

View/request a protocol for this paper from *Bio-protocol*.

## References

[1] R. E. Blankenship, *Molecular Mechanisms of Photosynthesis* (Blackwell Scientific, 2021).

[2] M. F. Hohmann-Marriot, R. E. Blankenship, Evolution of photosynthesis. Annu. Rev. Plant Biol. 62, 515–548 (2011). 21438681 10.1146/annurev-arplant-042110-103811

[3] C. J. Gisriel, C. Azai, T. Cardona, Recent advances in the structural diversity of reaction centers. Photosynth. Res. 149, 329–343 (2021). 34173168 10.1007/s11120-021-00857-9 PMC8452559

[4] D. A. Bryant, D. P. Canniffe, How nature designs light-harvesting antenna systems: Design principles and functional realization in chlorophototrophic prokaryotes. J. Phys. B At. Mol. Opt. Phys. 51, 033001 (2018).

[5] R. Croce, H. van Amerongen, Light harvesting in oxygenic photosynthesis: Structural biology meets spectroscopy. Science 369, eaay2058 (2020). 32820091 10.1126/science.aay2058

[6] D. A. Bryant, The molecular biology of cyanobacteria, in *Advances in Photosynthesis and Respiration, Vol. 1* (Springer, 1994).

[7] W. A. Sidler, Phycobilisome and phycobiliprotein structures, in *Advances in Photosynthesis and Respiration, Vol. 1*, *The Molecular Biology of Cyanobacteria*, Ed. D. A. Bryant (Springer, 1994), pp. 139–216.

[8] H. Liu, H. Zhang, D. M. Niedzwiedzki, M. Prado, G. He, M. L. Gross, R. E. Blankenship, Phycobilisomes supply excitations to both photosystems in a megacomplex in cyanobacteria. Science 342, 1104–1107 (2013). 24288334 10.1126/science.1242321 PMC3947847

[9] D. A. Bryant, G. Guglielmi, N. T. de Marsac, A.-M. Castets, G. Cohen-Bazire, The structure of cyanobacterial phycobilisomes: A model. Arch. Microbiol. 123, 113–127 (1979).

[10] L. Zheng, Z. Zheng, X. Li, G. Wang, K. Zhang, P. Wei, J. Zhao, N. Gao, Structural insight into the mechanism of energy transfer in cyanobacterial phycobilisomes. Nat. Commun. 12, 5497 (2021). 34535665 10.1038/s41467-021-25813-y PMC8448738

[11] M. A. Domínguez-Martín, P. V. Sauer, H. Kirst, M. Sutter, D. Bína, B. J. Greber, E. Nogales, T. Polívka, C. A. Kerfeld, Structures of a phycobilisome in light-harvesting and photoprotected states. Nature 609, 835–845 (2022). 36045294 10.1038/s41586-022-05156-4

[12] M.-Y. Ho, N. T. Soulier, D. P. Canniffe, G. Shen, D. A. Bryant, Light regulation of pigment and photosystem biosynthesis in cyanobacteria. Curr. Opin. Plant Biol. 37, 24–33 (2017). 28391049 10.1016/j.pbi.2017.03.006

[13] F. Wang, M. Chen, Chromatic acclimation processes and their relationships with phycobiliprotein complexes. Microorganisms 10, 1562 (2022). 36013980 10.3390/microorganisms10081562 PMC9415938

[14] M. Watanabe, D. A. Semchonok, M. T. Webber-Birungi, S. Ehira, K. Kondo, R. Narikawa, M. Ohmori, E. J. Boekema, M. Ikeuchi, Attachment of phycobilisomes in an antenna—Photosystem I supercomplex of cyanobacteria. Proc. Natl. Acad. Sci. U.S.A. 111, 2512–2517 (2014). 24550276 10.1073/pnas.1320599111 PMC3932850

[15] Y. Li, Y. Lin, C. J. Garvey, D. Birch, R. W. Corkery, P. C. Loughlin, H. Scheer, R. D. Willows, M. Chen, Characterization of red-shifted phycobilisomes isolated from the chlorophyll *f*-containing cyanobacterium *Halomicronema hongdechloris*. Biochim. Biophys. Acta Bioenerg. 1857, 107–114 (2016). 10.1016/j.bbabio.2015.10.009 26514405

[16] M.-Y. Ho, F. Gan, G. Shen, D. A. Bryant, Far-red light photoacclimation (FaRLiP) in *Synechococcus* sp. PCC 7335. II. Characterization of phycobiliproteins produced during acclimation to far-red light. Photosynth. Res. 131, 187–202 (2017). 27623780 10.1007/s11120-016-0303-5

[17] N. Soulier, T. N. Laremore, D. A. Bryant, Characterization of cyanobacterial allophycocyanins absorbing far-red light. Photosynth. Res. 145, 189–207 (2020). 32710194 10.1007/s11120-020-00775-2

[18] N. Soulier, K. Walters, T. N. Laremore, G. Shen, J. H. Golbeck, D. A. Bryant, Acclimation of the photosynthetic apparatus to low light in a thermophilic *Synechococcus* sp. strain. Photosynth. Res. 153, 21–42 (2022). 35441927 10.1007/s11120-022-00918-7

[19] F. Gan, S. Zhang, N. C. Rockwell, S. S. Martin, J. C. Lagarias, D. A. Bryant, Extensive remodeling of a cyanobacterial photosynthetic apparatus in far-red light. Science 345, 1312–1317 (2014). 25214622 10.1126/science.1256963

[20] F. Gan, D. A. Bryant, Adaptive and acclimative responses of cyanobacteria to far-red light. Environ. Microbiol. 17, 3450–3465 (2015). 26234306 10.1111/1462-2920.12992

[21] N. Soulier, D. A. Bryant, The structural basis of far-red light absorbance by allophycocyanins. Photosynth. Res. 147, 11–26 (2021). 33058014 10.1007/s11120-020-00787-y

[22] G. Shen, H. S. Leonard, W. M. Schluchter, D. A. Bryant, CpcM post-translationally methylates asparagine-71/72 of phycobiliprotein beta subunits in *Synechococcus* sp. PCC 7002 and *Synechocystis* sp. PCC 6803. J. Bacteriol. 190, 4808–4817 (2008). 18469097 10.1128/JB.00436-08 PMC2447021

[23] T. Schirmer, R. Huber, M. Schneider, W. Bode, M. Miller, M. L. Hackert, Crystal structure analysis and refinement at 2.5 Å of hexameric C-phycocyanin from the cyanobacterium *Agmenellum quadruplicatum*: The molecular model and its implications for light-harvesting. J. Mol. Biol. 188, 651–676 (1986). 3090271 10.1016/s0022-2836(86)80013-4

[24] P.-P. Peng, L. L. Dong, Y. F. Sun, X. L. Zeng, W. L. Ding, H. Scheer, X. Yang, K. H. Zhao, The structure of allophycocyanin B from *Synechocystis* PCC 6803 reveals the structural basis for the extreme redshift of the terminal emitter in phycobilisomes. Acta Crystallogr. Sect. D 70, 2558–2569 (2014). 25286841 10.1107/S1399004714015776 PMC8494197

[25] P. Cao, D. Cao, L. Si, X. Su, L. Tian, W. Chang, Z. Liu, X. Zhang, M. Li, Structural basis for energy and electron transfer of the photosystem I–IsiA–Flavodoxin supercomplex. Nat. Plants 6, 167–176 (2020). 32042157 10.1038/s41477-020-0593-7

[26] N. V. Karapetyan, Y. V. Bolychevtseva, N. P. Yurina, I. V. Terekhova, V. V. Shubin, M. Brecht, Long-wavelength chlorophylls in photosystem I of cyanobacteria: Origin, localization, and functions. Biochemistry (Moscow) 79, 213–220 (2014). 24821447 10.1134/S0006297914030067

[27] V. Mascoli, L. Bersanini, R. Croce, Far-red absorption and light-use efficiency trade-offs in chlorophyll *f* photosynthesis. Nat. Plants 6, 1044–1053 (2020). 32661277 10.1038/s41477-020-0718-z

[28] J. Zivanov, T. Nakane, B. O. Forsberg, D. Kimanius, W. J. H. Hagen, E. Lindahl, S. H. W. Scheres, New tools for automated high-resolution cryo-EM structure determination in RELION-3. eLife 7, e42166 (2018). 30412051 10.7554/eLife.42166 PMC6250425

[29] S. Q. Zheng, E. Palovcak, J. P. Armache, K. A. Verba, Y. Cheng, D. A. Agard, MotionCor2: Anisotropic correction of beam-induced motion for improved cryo-electron microscopy. Nat. Methods 14, 331–332 (2017). 28250466 10.1038/nmeth.4193 PMC5494038

[30] A. Rohou, N. Grigorieff, CTFFIND4: Fast and accurate defocus estimation from electron micrographs. J. Struct. Biol. 192, 216–221 (2015). 26278980 10.1016/j.jsb.2015.08.008 PMC6760662

[31] P. Emsley, B. Lohkamp, W. G. Scott, K. Cowtan, Features and development of Coot. Acta Crystallogr. Sect. D Biol. Crystallogr. 66, 486–501 (2010). 20383002 10.1107/S0907444910007493 PMC2852313

[32] E. F. Pettersen, T. D. Goddard, C. C. Huang, G. S. Couch, D. M. Greenblatt, E. C. Meng, T. E. Ferrin, UCSF Chimera—A visualization system for exploratory research and analysis. J. Comput. Chem. 25, 1605–1612 (2004). 15264254 10.1002/jcc.20084

[33] N. Guex, M. C. Peitsch, T. Schwede, Automated comparative protein structure modeling with SWISS-MODEL and Swiss-PdbViewer: A historical perspective. Electrophoresis 30, S162–S173 (2009). 19517507 10.1002/elps.200900140

[34] P. D. Adams, P. V. Afonine, G. Bunkóczi, V. B. Chen, I. W. Davis, N. Echols, J. J. Headd, L. W. Hung, G. J. Kapral, R. W. Grosse-Kunstleve, A. J. McCoy, N. W. Moriarty, R. Oeffner, R. J. Read, D. C. Richardson, J. S. Richardson, T. C. Terwilliger, P. H. Zwart, PHENIX: A comprehensive python-based system for macromolecular structure solution. Acta Crystallogr. Sect. D Biol. Crystallogr. 66, 213–221 (2010). 20124702 10.1107/S0907444909052925 PMC2815670

[35] P. V. Afonine, B. K. Poon, R. J. Read, O. V. Sobolev, T. C. Terwilliger, A. Urzhumtsev, P. D. Adams, Real-space refinement in PHENIX for cryo-EM and crystallography. Acta Crystallogr. Sect. D 74, 531–544 (2018). 10.1107/S2059798318006551 PMC6096492 29872004

[36] J. J. Snellenburg, S. Laptenok, R. Seger, K. M. Mullen, I. H. M. van Stokkum, Glotaran: A Java-based graphical user interface for the R package TIMP. J. Stat. Softw. 49, 1–22 (2012).

[37] A. V. Digris, E. G. Novikov, V. V. Skakun, V. V. Apanasovich, “Global analysis of time-resolved fluorescence data,” in *Fluorescence Spectroscopy and Microscopy: Methods and Protocols*, Y. Engelborghs, A. J. W. G. Visser, Eds. (Humana Press, 2014), pp. 257–277. 10.1007/978-1-62703-649-8_10 24108629

[38] S. J. A. van Gisbergen, J. G. Snijders, E. J. Baerends, Implementation of time-dependent density functional response equations. Comput. Phys. Commun. 118, 119–138 (1999).

[39] G. te Velde, F. M. Bickelhaupt, E. J. Baerends, C. Fonseca Guerra, S. J. A. van Gisbergen, J. G. Snijders, T. Ziegler, Chemistry with ADF. J. Comput. Chem. 22, 931–967 (2001).

[40] A. B. Doust, C. N. J. Marai, S. J. Harrop, K. E. Wilk, P. M. G. Curmi, G. D. Scholes, Developing a structure–function model for the cryptophyte phycoerythrin 545 using ultrahigh resolution crystallography and ultrafast laser spectroscopy. J. Mol. Biol. 344, 135–153 (2004). 15504407 10.1016/j.jmb.2004.09.044

[41] R. R. Sonani, G. D. Gupta, D. Madamwar, V. Kumar, Crystal structure of allophycocyanin from marine cyanobacterium *Phormidium* sp. A09DM. PLOS ONE 10, e0124580 (2015). 25923120 10.1371/journal.pone.0124580 PMC4414346

[42] J. M. Womick, A. M. Moran, Exciton coherence and energy transport in the light-harvesting dimers of allophycocyanin. J. Phys. Chem. B 113, 15747–15759 (2009). 19894754 10.1021/jp907644h

[43] A. D. Becke, Density-functional thermochemistry. III. The role of exact exchange. J. Chem. Phys. 98, 5648–5652 (1993).

[44] C. Lee, W. Yang, R. G. Parr, Development of the Colle-Salvetti correlation-energy formula into a functional of the electron density. Phys. Rev. B 37, 785–789 (1988). 10.1103/physrevb.37.785 9944570

[45] P. J. Stephens, F. J. Devlin, C. F. Chabalowski, M. J. Frisch, Ab initio calculation of vibrational absorption and circular dichroism spectra using density functional force fields. J. Phys. Chem. 98, 11623–11627 (1994).

[46] C. Curutchet, V. I. Novoderezhkin, J. Kongsted, A. Muñoz-Losa, R. van Grondelle, G. D. Scholes, B. Mennucci, Energy flow in the cryptophyte PE545 antenna is directed by bilin pigment conformation. J. Phys. Chem. B 117, 4263–4273 (2013). 22992117 10.1021/jp305033d

[47] Y. Ren, B. Chi, O. Melhem, K. Wei, L. Feng, Y. Li, X. Han, D. Li, Y. Zhang, J. Wan, X. Xu, M. Yang, Understanding the electronic energy transfer pathways in the trimeric and hexameric aggregation state of cyanobacteria phycocyanin within the framework of Förster theory. J. Comput. Chem. 34, 1005–1012 (2013). 23299487 10.1002/jcc.23221

[48] G. Cinque, R. Croce, R. Bassi, Absorption spectra of chlorophyll *a* and *b* in Lhcb protein environment. Photosynth. Res. 64, 233–242 (2000). 16228461 10.1023/A:1006467617697

[49] V. I. Novoderezhkin, R. Croce, M. Wahadoszamen, I. Polukhina, E. Romero, R. van Grondelle, Mixing of exciton and charge-transfer states in light-harvesting complex Lhca4. Phys. Chem. Chem. Phys. 18, 19368–19377 (2016). 27375175 10.1039/c6cp02225a

[50] F. Sievers, A. Wilm, D. Dineen, T. J. Gibson, K. Karplus, W. Li, R. Lopez, H. M. William, M. Remmert, J. Söding, J. D. Thompson, D. G. Higgins, Fast, scalable generation of high quality protein multiple sequence alignments using Clustal Omega. Mol. Syst. Biol. 7, 1–6 (2011). 10.1038/msb.2011.75 PMC3261699 21988835

[51] J. Jumper, R. Evans, A. Pritzel, T. Green, M. Figurnov, O. Ronneberger, K. Tunyasuvunakool, R. Bates, A. Žídek, A. Potapenko, A. Bridgland, C. Meyer, S. A. A. Kohl, A. J. Ballard, A. Cowie, B. Romera-Paredes, S. Nikolov, R. Jain, J. Adler, T. Back, S. Petersen, D. Reiman, E. Clancy, M. Zielinski, M. Steinegger, M. Pacholska, T. Berghammer, S. Bodenstein, D. Silver, O. Vinyals, A. W. Senior, K. Kavukcuoglu, P. Kohli, D. Hassabis, Highly accurate protein structure prediction with AlphaFold. Nature 596, 583–589 (2021). 34265844 10.1038/s41586-021-03819-2 PMC8371605

[52] V. Mariani, M. Biasini, A. Barbato, T. Schwede, lDDT: A local superposition-free score for comparing protein structures and models using distance difference tests. Bioinformatics 29, 2722–2728 (2013). 23986568 10.1093/bioinformatics/btt473 PMC3799472

