# Supplementary material for: Helical allophycocyanin nanotubes absorb far-red light in a thermophilic cyanobacterium
Source: Sci Adv. 2023 Mar 24;9(12):eadg0251. doi: 10.1126/sciadv.adg0251 (PMC10038336; doi:10.1126/sciadv.adg0251)
Supplement: Supplementary file 1 — Supplementary Texts S1 and S2 Figs. S1 to S16 Tables S1 and S2 References [file sciadv.adg0251_sm.pdf]

Science Advances
AAAS

Supplementary Materials for

Helical allophycocyanin nanotubes absorb far-red light in a thermophilic cyanobacterium

Christopher J. Gisriel *et al.*

Corresponding author: Christopher J. Gisriel, christopher.gisriel@yale.edu; Roberta Croce, r.croce@vu.nl; Gary W. Brudvig, gary.brudvig@yale.edu; Donald A. Bryant, dab14@psu.edu

Sci. Adv. **9**, eadg0251 (2023)
DOI: 10.1126/sciadv.adg0251

This PDF file includes:

Supplementary Texts S1 and S2
Figs. S1 to S16
Tables S1 and S2
References

Supplementary Text S1

Lack of excitonic interactions among PCBs and retrieval of parameters for modeling FRET in FRL-AP.

The use of Förster theory was deemed to be appropriate for modeling EET dynamics in FRL-AP helices because the absorbance peaks of the α and β-subunits (709 and 621 nm, respectively) are very far apart in energy (~2,000 cm^{-1}) and huge electronic coupling values would then be required in the system to induce any sizeable excitonic effects. For canonical AP trimers, whose nearest distance between α- and β-PCBs is very similar to that of FRL-AP helices, the site-energies of the two PCBs (15,300 and 16,060 cm^{-1}, respectively) and the electronic coupling between them (-163 cm^{-1}) has been determined using spectral modeling (*42*). In the framework of Frenkel excitons, this implies almost fully (96%) localized excited states. Assuming then that the electronic coupling between α/β-PCB pairs in the FRL-AP is on the same order of magnitude as in canonical AP, and that the gap in site-energies is at least twice as large, we can exclude excitonic effects and assume localized states.

Thus, the FRET rate (in ps^{-1}) can be calculated in the following way:

$$k_{D\to A} = 1.18\, V^2 J$$

The electronic coupling V (in cm^{-1}) is calculated according to the ideal dipole approximation:

$$V_{DA} = 5.04 \frac{\mu_D \mu_A \kappa}{R^3}$$

μ_D = Transition dipole moment (TDM) of the donor in Debye

μ_A = TDM of the acceptor in Debye

R = center-to-center separation between the donor and acceptor in nm

κ is the orientation factor and can be calculated as follows:

$$\kappa = \hat{\mu}_D \cdot \hat{\mu}_A - 3(\hat{\mu}_D \cdot \hat{R}_{DA})(\hat{\mu}_A \cdot \hat{R}_{DA})$$

$\hat{\mu}_D$ = Unit vector of the TDM of the donor

$\hat{\mu}_A$ = Unit vector of the TDM of the acceptor

$\hat{R}_{DA}$ = Unit vector of the center-to-center separation of the donor and acceptor

J represents the spectral overlap integral for the fluorescence of the donor and the absorption of the acceptor in which both spectra are normalized to an area of 1 on a cm^{-1} scale:

$$J_{DA} = \int_0^\infty F_D(\nu) A_A(\nu)\, d\nu$$

The orientation of the TDM for the S_1 state of PCB is not as well established in the literature as it is for some other chromophores (e.g., Chl molecules). For the closely related chromophore phycoerythrobilin (PEB), the TDM orientation was calculated using a CIS/3-21g level of theory to be oriented roughly along the CHD to the C4A atom (these refer to the atom names in the coordinate files for PCBs) (*40*). In another recent work in which the EET dynamics of the cyanobacterial PBS was modeled using Förster theory, the TDM was taken to be oriented along the axis of the conjugated parts of the bilins (*11*). We have calculated the orientation of the TDM for the α-PCB and β-PCBs in our system using a TD-DFT B3LYP-D3BJ/TZ2P level of theory (*43–45*) as described in **Materials and Methods**, which produces similar results. The calculated orientations of the S_1 TDM of the α-PCB and β-PCB are shown in **fig. S11**. The calculated TDMs are almost parallel to the axis connecting the NA and the NB atom in the molecules, and this axis was therefore used for the FRET calculations. The center-to-center separation was determined as the axis connecting the CHA atoms of the donor and acceptor molecules.

Several values have been reported for the magnitude of the S_1 TDM of bilins. In the EET modeling of a cyanobacterial PBS a value of 15 D was used for AP chromophores (*11*). Quantum mechanical calculations of the S_1 TDM magnitudes of the bilins in the PE545 complex, which includes PEB and 15,16-dihydrobiliverdin, yielded magnitudes in the range of ~10-13.8 D in two different studies (*40, 46*). In another study, quantum mechanical calculations were performed to retrieve the S_1 TDM magnitudes of the PCB pigments found in C-phycocyanin and magnitudes in the range of 11.1-13.5 D were found (*47*). Spectral modeling of the C-phycocyanin complex yielded transition dipole moments for the pigments in the range of 5.4-6.5 D. Overall, we therefore assume that the TDM magnitude for the PCBs in FRL-AP are within the range 5.4-15 D (*47*). We have therefore chosen to use a TDM magnitude that yields results that are closest to the results obtained with the pump-probe experiment, which is 10 D.

Finally, the spectral overlap of the α-PCB and β-PCB is required to calculate FRET rates, for which the emission and absorption spectrum of both are required. For the α-PCB emission spectrum, we used the steady-state emission spectrum of the FRL-AP. This is appropriate because the pump-probe experiment showed that excited-state decay arises only from α-subunits, and because the site-energies of the α- and β-PCBs are at least 1,800 cm^{-1} apart (~9 k_BT at room temperature). Thus, the steady-state emission spectrum of the FRL-AP contains contributions from only the α-PCB. To obtain the steady-state emission spectrum of the β-PCB, we subtracted the α-PCB emission spectrum (recombinantly expressed and isolated from *Synechococcus* 7002) from the emission spectrum of FRL-AP containing a fraction of free β-subunits (recombinantly expressed and isolated from *E. coli*). The steady-state emission spectrum of the α- and β-subunits are shown in **fig. S12A**.

To obtain the absorption spectra of the α and β PCB chromophores, we have fitted to FRL-AP the absorption spectrum with the reversed emission spectra of the α-PCB and β-PCBs (**fig. S12B**), allowing the peak positions and relative amplitudes to vary. It was found that in the β-PCB absorption region the reversed disconnected β-PCB emission spectrum was not wide enough to correctly fit the data. Therefore, as an additional parameter the width of the β-PCB absorption was allowed to vary. The β-PCB emission spectrum that was used for the spectral overlap calculations was then obtained by reversing the fitted β-PCB absorption spectrum while considering a Stokes shift of 5 nm (which is the same as the determined Stokes shift of the α-PCB). The fitted absorbance spectrum is shown in **fig. S12B**. The area normalized spectra used for the spectral overlap calculations are shown in **fig. S12C**.

Using the above-mentioned parameters, formulas, and the FRL-AP structure, the Förster rates between all the pigments in the system could be calculated. Given the initial distribution of excited state populations for the pigments, the time-evolution of these populations could be calculated by solving the following master equation:

$$\frac{dp_m(t)}{dt} = \sum_{n\neq m} p_n(t)\cdot k_{n\to m} - p_m(t)\cdot\left\{\left(\sum_{n\neq m} k_{m\to n}\right) + \frac{1}{\tau_d}\right\}$$

$P_m(t)$ = excited-state population of pigment m as a function of time t
τ_d = excited-state lifetime of pigment m

The excited-state lifetime τ_d of each pigment in the FRL-AP system was set to the value as determined by the time-resolved absorption experiment (889 ps, **Fig. 6**).

To simulate the EET dynamics of the time-resolved absorption experiment, the master equation was solved for the initial conditions in which the initial excitation distribution was equally divided over all the β-PCBs as described in the main text. The total simulated time evolution for

the excitation densities on the α- and β-PCBs is shown in **fig. S13A**. Using a TDM magnitude for the PCBs of 10 D, the simulation nicely matches the experimental data. We also calculated the time in which energy is transferred along a 13-protomer nanotube. The calculated time-evolution of the excited-state populations of the α-PCBs in the system upon initial excitation of the W α-PCB is shown in **fig. S13B**.

Supplementary Text S2

EET efficiency in FRL-AP nanotubes.

We have evaluated the overlap integrals for EET from α-PCBs to typical Chls *a* and to PSI red-form Chls (**fig. S15**). In order to do so, we additionally needed to determine the absorption spectra of Chl *a* and of the PSI red-form Chls. For the absorption spectrum of Chl *a*, the determined spectrum of Chl *a* in an Lhcb environment was taken from Cinque et al. (*48*) and shifted to 680 nm. The absorption spectrum for a PSI red-form Chl was modeled as a charge-transfer state and was taken from Novoderezhkin et al. (*49*). The peak of the spectrum was then shifted to 710 nm. Considering the proposed positioning of the FRL-AP with respect to PSI, we imagine that the energy must be extracted near the bottom of a nanotube. To estimate the upper limit of the FRL-AP antenna efficiency, we connected one of the bottom α-PCB chromophores to a perfect trap, i.e., a bottom α-PCB transfers energy with an infinite rate to the trap and there is no back-transfer. For a situation in which a single α-PCB is initially excited, we can calculate the transient population of the trap using our FRET model. The final population of the trap then represents the efficiency of EET for the given initial condition. By averaging over all α-PCB chromophores that can be individually initially excited, we find the total maximal EET efficiency of FRL-AP (**fig. S15**). We have considered three locations for the trap: E, G or A (the isolated α-PCB). Their calculated total maximal EET efficiencies are 40%, 46% and 32%, respectively. If we consider a FRL-AP nanotube with half the length (only PCBs N-Z are present in the system), and the trap at S, U and O, then we find EET efficiencies of 65%, 65% and 57%, respectively.

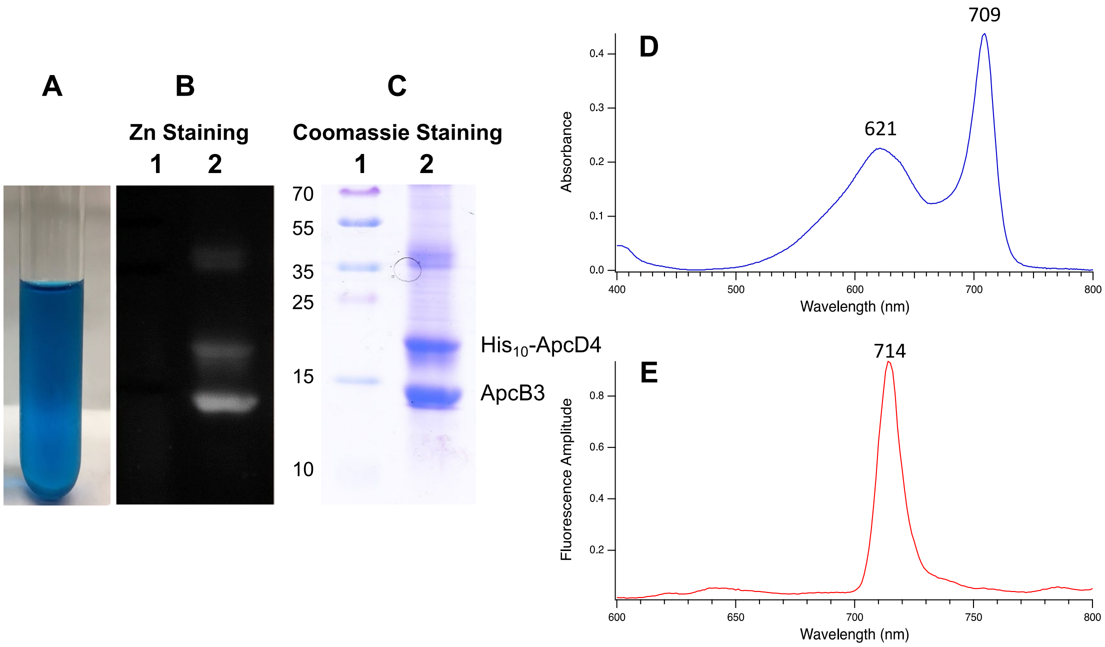

Fig. S1. Purification and characterization of FRL-AP. (**A**) Solution of purified FRL-AP (concentration ~10 mg mL^{-1}). (**B**) and (**C**), SDS-PAGE of purified FRL-AP. Lane 1 contains molecular mass standards. Masses in kDa are indicated in (C). Lane 2 contains 10 µg of FRL-AP stained with Zn^{2+} in (B) or Coomassie Blue in (C). The bands of greatest intensity correspond to His-tagged ApcD4 and ApcB3, respectively, as labeled. The weak bands at ~36-38 kDa correspond to minor populations of dimeric ApcD4-ApcB3 due to incomplete dissociation. (**D**) Absorbance spectrum of purified helical FRL-AP. (**E**) 77 K fluorescence emission spectrum of purified FRL-AP. The excitation wavelength was 590 nm. In (D) and (E), peaks are labeled in units of nm. These differ from the absorbance maxima of AP (λ_{max} = ~650 nm and 617 nm); see Fig. 4B or S10C) and AP-B (λ_{max} = ~670 nm and 617 nm; see Fig. 4B or S10C), which have their fluorescence emission maxima at about 660 nm and 680 nm, respectively (*4, 7*).

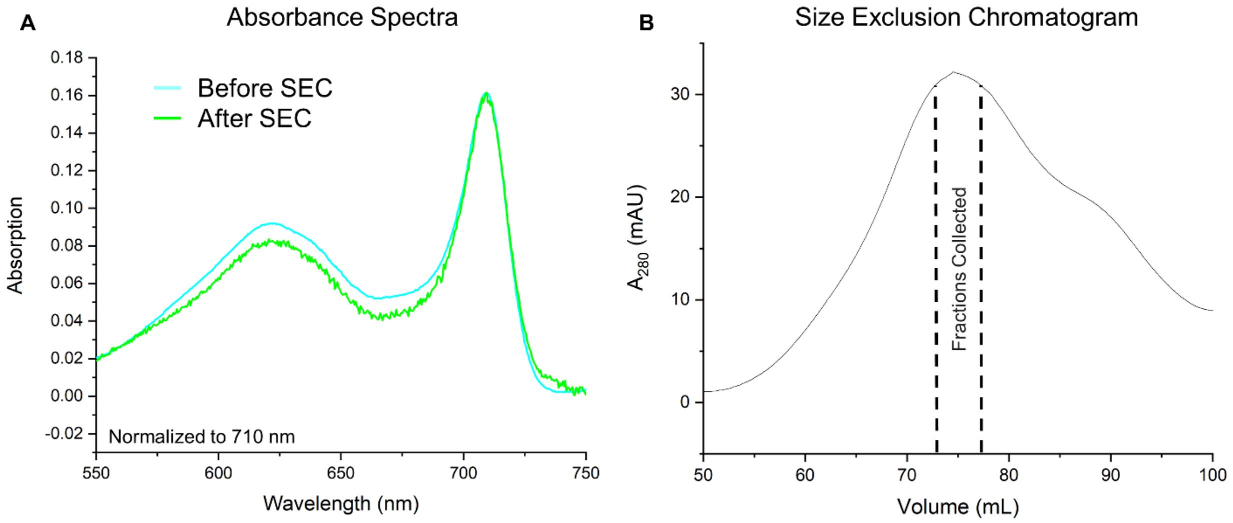

Fig. S2. Size exclusion chromatography of FRL-AP. (**A**) Absorbance spectra before and after size exclusion chromatography. The slightly decreased peak at ~620 nm after size exclusion chromatography (SEC) is likely due to the removal of some protomers that are not found in oligomers. Spectra were normalized at 710 nm for improved comparison. (**B**) Size exclusion chromatography chromatogram. A wide distribution of sizes was observed from which fractions were used for cryo-EM.

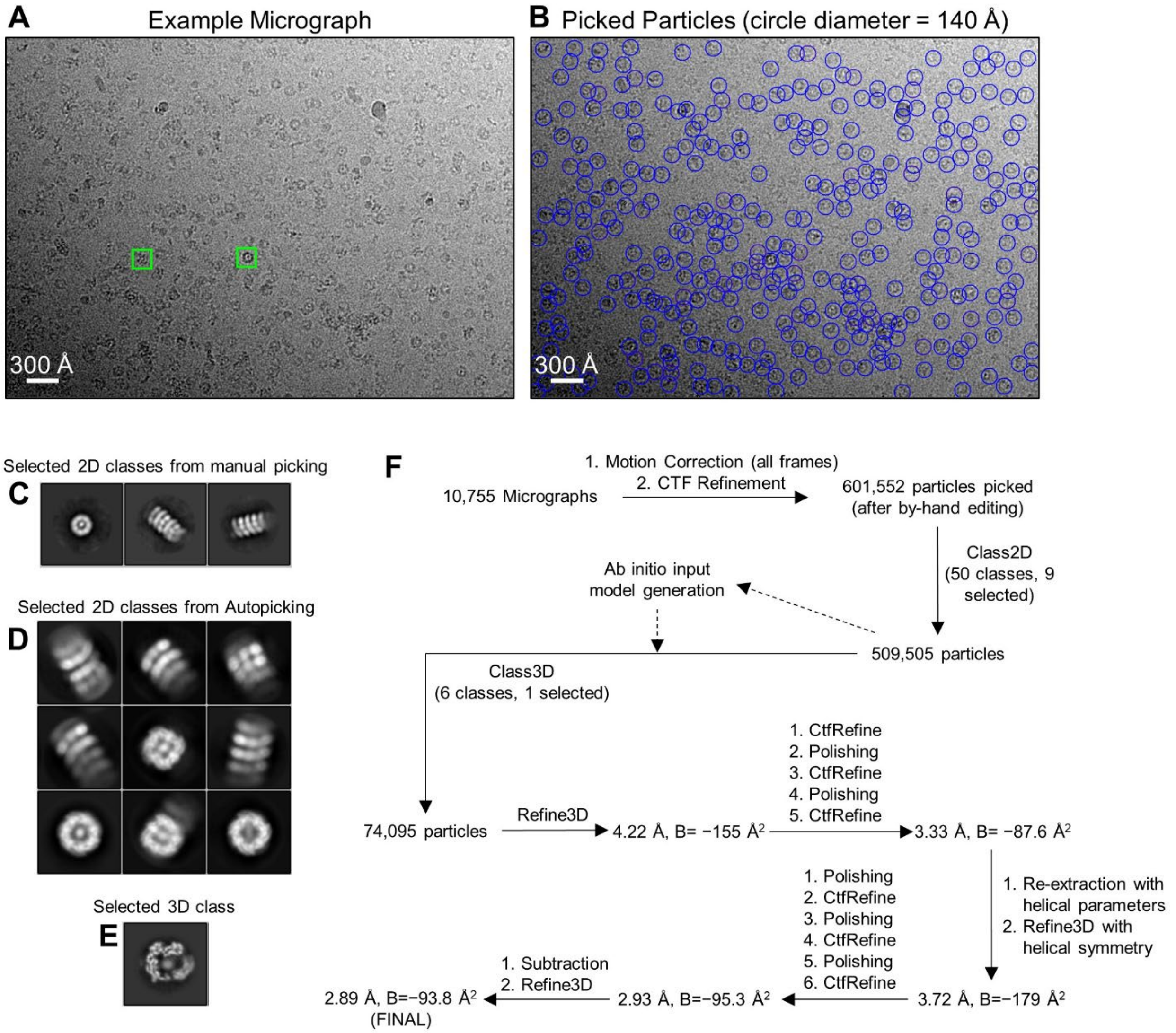

Fig. S3. Cryo-EM data processing. (**A**) Example micrograph collected during data collection. One side view of a short helix and one top view are shown with green boxes. (**B**) Identical micrograph as (A), but with selected autopicked particles. (**C**) 2D classes selected after manually picking ~780 particles. (**D**) Selected 2D classes after autopicking. (**E**) Selected 3D class. (**F**) Workflow of data processing in RELION 3.1 (*28*).

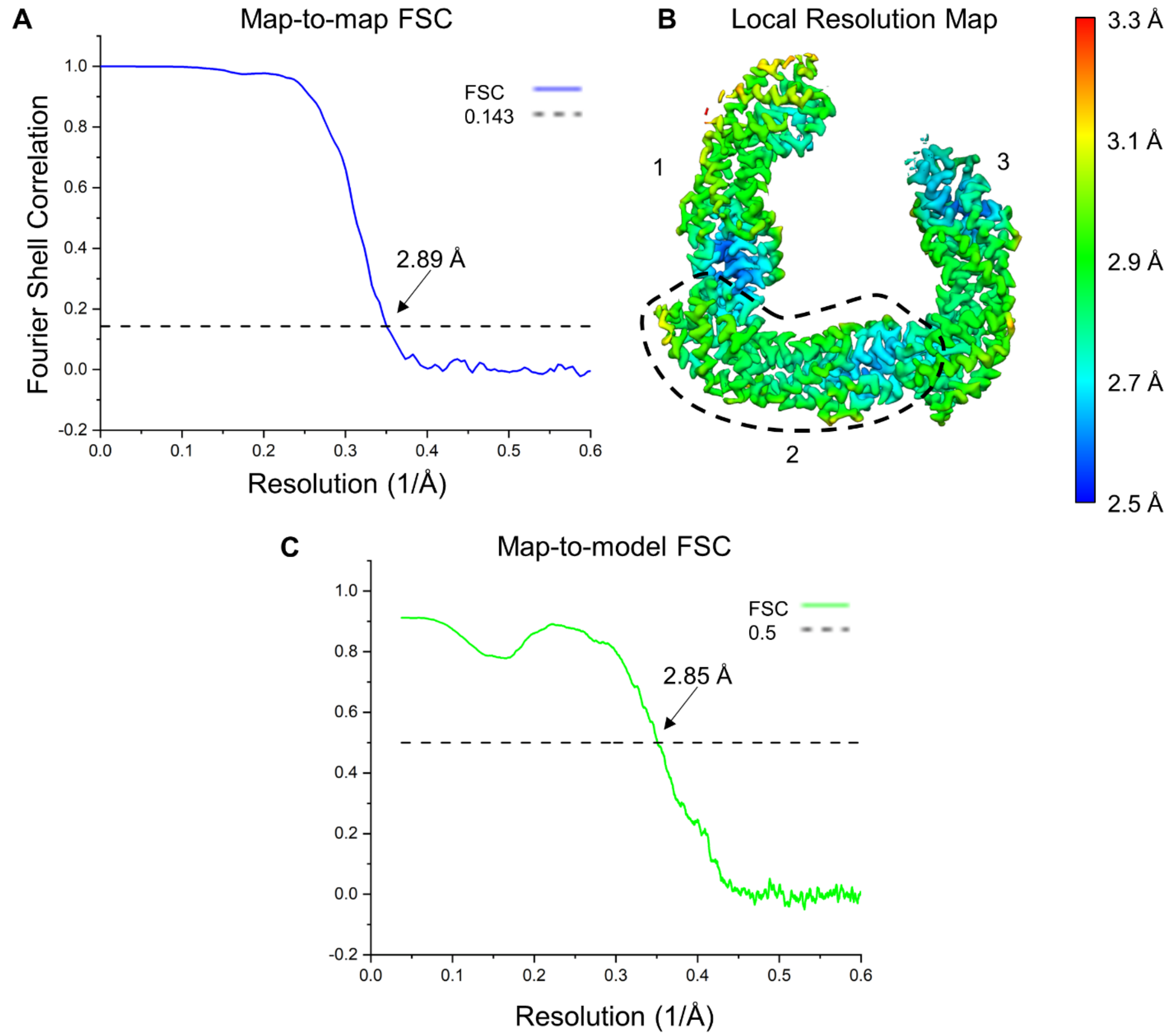

Fig. S4. Cryo-EM data resolution. (**A**) Map-to-map Fourier shell correlation calculated by RELION 3.1 (*28*). (**B**) Local resolution of a central three protomers in the helical map. The three protomers are numbered and the central one is outlined with a dotted line for reference. **c**, Map-to-model Fourier shell correlation calculated by Phenix (*34, 35*).

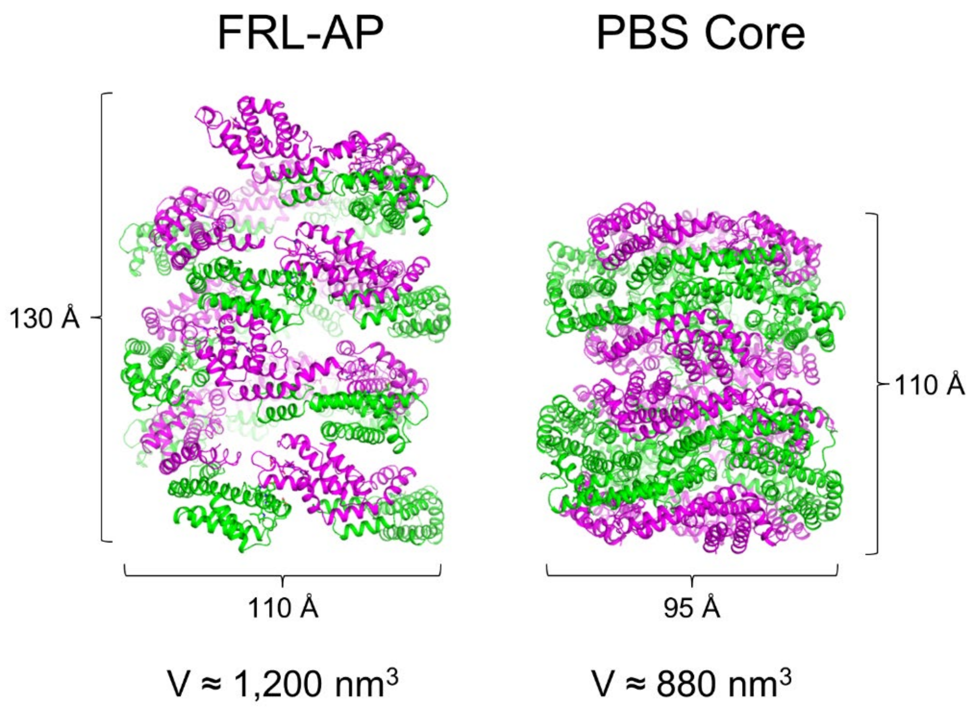

Fig. S5. Comparison of FRL-AP and a PBS core. 12 protomers of a FRL-AP helical nanotube are compared to a PBS core comprising 12 protomers (a stack of four AP trimers and related complexes) from *Synechococcus* sp. PCC 7002 (PDB 7EXT) (*10*). α-subunits are colored green and β-subunits are colored magenta. The length, diameter, and approximate volumes of each are labeled.

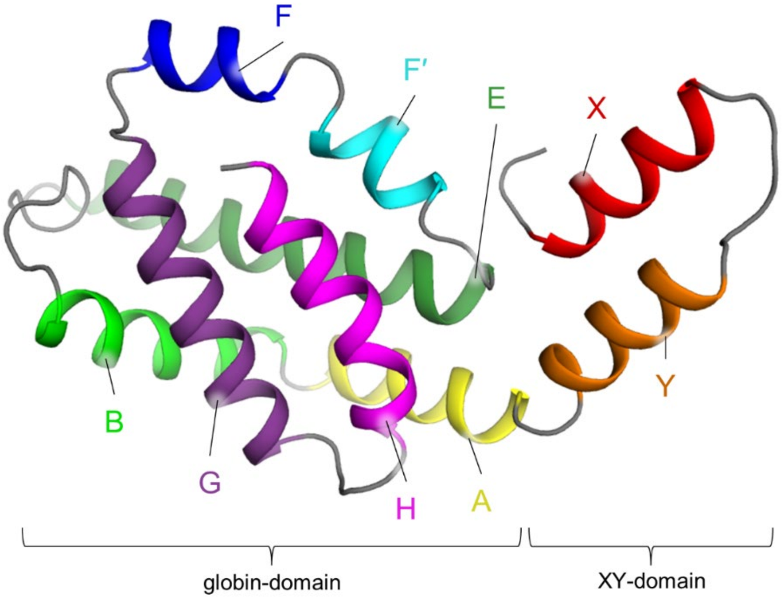

Fig. S6. Secondary structure nomenclature of PBPs used herein. The α-subunit of FRL-AP is shown with helices colored and labeled according to the nomenclature defined previously (*23*). This nomenclature is maintained throughout the manuscript for all discussions of PBPs.

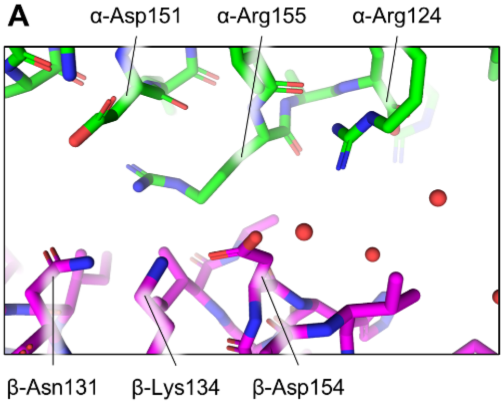

B

α-subunit
```
FRL-AP  (ApcD4) VPLPNLRVAMSCLKEVAAGILSSEEMALAAPYFDRLIRAF- 157
AP-B    (ApcD1) VPVPGMVDAVTVLKDAALGLLSAEDANETAPYFDYIIQFMS 161
AP      (ApcA1) TPIPAVAEAVRCMKSVASSLLSGENAAEAASYFDYVVGAMQ 153
                .*:* :   *:   :*..* .:**.*:    :* *** ::   :
```

β-subunit
```
FRL-AP  (ApcB3) VPIDRVIQALNAMKEVLTESLDTEASQEMAVYLDHIIAGLS 161
AP-B    (ApcB1) VPISSTVQAIQAIKEVTASLVGADAGKEMGVYLDYICSGLS 161
AP      (ApcB1) VPVGATVQAIQAMKEVTATLVGADAGKEMGVYFDY------ 155
                **:. .:**::*:*** :  :.::*.:**.**:*:
```

Fig. S7. Interactions in the FRL-AP structure between helix H of an α-subunit and helix H of the β-subunit in the helical level above it. (**A**) The interface of interest, showing both subunits in stick representation. The nearest charged or polar uncharged residues that may participate in H-bonding interactions are labeled. (**B**) Partial sequence alignment of FRL-AP, AP-B, and AP. Residues in bold correspond to the labels in panel (A). Clustal Omega sequence conservation identifiers are shown below the alignment for each position.

A

α-subunits (D4, D2, D3, D5, D1, and A1)

```
                                                          llXXXXXXXXXXXllllllYYYYYYYYYYYllAAAAAAAAAAAAAlBBBB
ApcD4 Synechococcus sp. A1463 (WP_099812040.1)            ----------MSIVAQVIAQSDAADRFLSSAEIAKLEDFFSKGQVRIRAAQKLAENEQKI   50
ApcD4 Chroococcidiopsis sp. FACHB-1243 (WP_192159074.1)   ----------MSLVAQVIAQSDEADRFLSRTELDKLQDFFKTGETRLKVAQILSQNEQKI   50
ApcD4 Chroococcidiopsis thermalis PCC 7203 (WP_015156293.1) ----------MSLVAQVIAQSDEADRFLSRTELDKLQDFFKTGETRLKVAQILSKNEQKI   50
ApcD4 Gloeocapsa sp. PCC 7428 (WP_015190166.1)           ----------MSIVAQVIAQSDDANRFLSNTELDKLQDFFRTGEQRLKVAQILTQNEQKI   50
ApcD4 Chlorogloeopsis sp. PCC 9212 (WP_016874155.1)      ----------MSIVAQVIAQSDDAARFLSRTELDKLDNFFKSGETRLRVAQILAQNEQNI   50
ApcD4 Halomicronema hongdechloris (WP_080809693.1)       ----------MSIVAKVIAQSDEANRFLSSAELNKLQDFFKDGAVRISAAQKLAANQQKL   50
ApcD4 Leptolyngbya sp. PCC 6406 (WP_008312402.1)         ----------MSIVAKVIAQSDEASRFLSSAELTKLQDFFGNGTVRISASQKLASNQQKI   50
ApcD4 Xenococcus sp. PCC 7305 (WP_006512043.1)           ----------MSIVAKVIAQSDRSDRFPSTAEITQLQDFFNNSSVRISTAQKLSENQQKI   50
ApcD4 Pleurocapsa sp. CCALA 161 (WP_106232622.1)        ----------MSIVAKVIAQSDMSDRFLSSAELTQLQDFFNKGGVRISAAQKLAANEQKI   50
ApcD4 Synechococcus sp. PCC 7335 (WP_006454943.1)       ----------MSIVAKAIAQSDRSDRFLGSTELTQLQDFFNNSSARISAAQKLAANQQKI   50
ApcD4 Romeria gracilis (WP_193906454.1)                 ----------MSIVAKVIAQSDRADRFLSSAELTQLQDFFNDSGARISAAQKLAANQQKI   50
ApcD4 Gloeomargarita lithophora (WP_071455395.1)        ----------MSLVAQVIAQSDAAERFLSSTELNKLAEFFSKGALRVRVAEKLAANEKKI   50
ApcD2 Synechococcus sp. PCC 7335 (WP_006455320.1)       ----------MSVISQVIATADREVRYLSKGELDAINRFFNNGPQRLRIVSILNSNAEEI   50
ApcD2 Leptolyngbya sp. JSC-1 (IMG: 2022833634)         ----------MSIITNVIATADREARYLNVEELNAVQNFYAAGRDRLRLAATLAANEQRI   50
ApcD2 Chroococcidiopsis thermalis PCC 7203 (WP_015153115.1) ----------MSIITKAIASADREARYLSPGELRTIRDFYNGGENRLRIATTLIENRKEI   50
ApcD2 Calothrix sp. PCC 7507 (WP_015126588.1           ----------MSIITKSIASADREARYLSPGELNAIRDFYEGGFYRMRIAITLTENEQKI   50
ApcD2 Chlorogloeopsis sp. PCC 9212 (WP_016873422.1)     ----------MSIIIKSIVNADREARYLNAGELRAIQEFYENGVSRLNFAVTLTENEKTI   50
ApcD2 Fischerella thermalis PCC 7521 (WP_009453700.1)  ----------MSIIIKSIVNADREARYLNAGELSAIQEFYESGVSRLNLAMTLTENEQKI   50
ApcD3 Leptolyngbya sp. JSC-1 (MBF2050150.1)           ----------MSLVKQVIQNADEELRYPTPGEIRMIQNFCQTGERRIQIAQKLAAAEQDL   50
ApcD3 Calothrix sp. PCC 7507 (WP_015126586.1)         ----------MSLVKQVILNADEELRYPTPAEIRMIQNFCHSGEKRIRIATTLAKNQNRL   50
ApcD3 Chlorogloeopsis sp. PCC 9212 (WP_016873424.1)     ----------MSLVKQVIENADEQMRYPTPGEIRMIQKFCHSGDKRIRIATVLASNQNRL   50
ApcD3 Fischerella thermalis PCC 7521 (WP_009453698.1)  ----------MSLVKQVIENADEQMRYPSPGEIRMIQNFCHSGDKRIRIATTLASNQNRL   50
ApcD3 Synechococcus sp. PCC 7335 (WP_006456515.1)       ----------MSIVKQIISNADEELRYPTPGELEMIRSFCKTGASQIQLAKTLESHAPTI   50
ApcD3 Chroococcidiopsis thermalis PCC 7203 (WP_015153117.1) ----------MSIVKQMILNADEEVRYLTPGEIHALQNFYRSGTERIRLAKVLAQNEKKI   50
ApcD5 Synechococcus sp. PCC 7335 (WP_006456289.1)       ----------MSLVTELILSADSEARYPAPKELRIFQDFVKTGEQRVRIAKALAANEERI   50
ApcD5 Chroococcidiopsis thermalis PCC 7203 (WP_015153113.1) ----------MSIVTELILNADSESRYPAPKEIQVYQNFVKTGEQRIRIAKILAENEQRI   50
ApcD5 Leptolyngbya sp. JSC-1 (IMG: 2022833632)         ----------MSVVTELILNADSESRYPVPKEMRIFQDFLRSGEQRIRIAATLAENEQRI   50
ApcD5 Calothrix sp. PCC 7507 (WP_015126590.1)         ----------MSIVTELILNADSESRYPAPKELRVFQEFLKSGDQRIRIAKILSQNEQQI   50
ApcD5 Chlorogloeopsis sp. PCC 9212 (WP_016873420.1)     ----------MSVVTELILNADSESRYPAPKELRIFQDFVKTGDQRIRIAKILSDNEQLI   50
ApcD5 Fischerella thermalis PCC 7521 (WP_009453702.1)  ----------MSVVTELILNADSESRYPAPKELRIFQDFLRTGDQRIRIAKILAENEQLI   50
ApcD1 Synechococcus sp. A1463 (WP_011431562.1)            ----------MSVINQIIETADDQLRYLSVSELQAIKDYMSSGEKRLQIAQVLTENKKRI   50
ApcD1 Synechocystis sp. PCC 6803 (WP_010871516.1)       ----------MSVVSQVILQADDQLRYPTSGELKGIQAFLTTGAQRIRIAETLAENEKKI   50
ApcD1 Synechococcus sp. PCC 7002 (WP_012307741.1)       ----------MSVVSQVILRADDELRYPSSGELSGIKNFLATGAVRIRIAEALADNEKKI   50
ApcD1 Leptolyngbya sp. PCC 7376 (WP_015135290.1)        ----------MSVVSQVILKADDELRYPSSGELTGMESFLATGAIRIRIAEALADSEKKI   50
ApcD1 Synechococcus sp. PCC 6301 (WP_011243585.1)       ----------MTIVSQVILKADDELRYPSGGELKNITDFFKTGEQRLRIAQVLSDSEKKI   50
ApcD1 Thermosynechococcus sp. CL-1 (QEQ1666.1)           MRDVLSGVSNMSVISQVLLKADDELRYPTTGELQTISDFFQTGEQRLRIATTLAENEKRI   60
ApcD1 Arthrospira platensis (GCE93786.1)                ----------MTVVSQVILKADDELRYPTTGELKNISDFLKTGEQRLRIVDTLTENEKKI   50
ApcD1 Synechococcus sp. PCC 7335 (WP_006454063.1)       ----------MSVVSQVILNADDELRYPTSGELKGIENFLKTGDQRMRIAQILADNEKKI   50
ApcD1 Leptolyngbya sp. JSC-1 (WP_036003919.1)           ----------MSVVTQVILNADEELRYPSSGELKSIQNFLQTGSQRMRIAATLADNEKKI   50
ApcD1 Chroococcidiopsis thermalis PCC 7203 (WP_015152396.1) ----------MSVVSQVILKADDELRYPSAGELENIKNFLQTGIQRMRIAATLAENEKKI   50
ApcD1 Nostoc sp. PCC 7524 (WP_015139163.1)              ----------MTVISQVILQADDELRYPSSGELKSIREFLQTGLQRTRIAGTLAENEKKI   50
ApcD1 Anabaena sp. PCC 7120 (WP_010997797.1)            ----------MTVISQVILQADDELRYPSSGELKSISDFLQTGVQRTRIVATLAENEKKI   50
ApcD1 Calothrix sp. PCC 7507 (WP_015130965.1)           ----------MTVISQVIFKADDELRYPSSGELKSIQNFLQTGIQRTRIAATLAENEKKI   50
ApcD1 Chlorogloeopsis fritschii PCC 9212 (WP_016878526.1) ----------MTVISQVILKADDELRYPSSGELKSIKDFLQTGEQRVRIANTLAENEKKI   50
ApcD1 Nostoc sp. PCC 7107 (WP_015113750.1)              ----------MTVISQVILKADDELRYPSSGELKSIKEFLQTGEQRTRIAATLAENEKKI   50
ApcA1 Anabaena sp. PCC 7120 (WP_010994626.1)            ----------MSLIIKSILNADAEARYFRPGELAQIKGFNASAASRLRLVQALTENRERI   50
ApcA1 Synechococcus sp. PCC 6301 (WP_011243498.1)       ----------MSIVSKSIVNADAEARYLSPGELERIKTFVVGGDRRLRIAQTIAESRERI   50
ApcA1 Synechococcus sp. PCC 7335 (WP_006456136.1)       ----------MSIVTKSIVNADAEARYLSPGELDRIKGFVTSGERRVRIAQVLTESRERI   50
ApcA1 Synechocystis sp. PCC 6803 (WP_010872503.1)       ----------MSIVTKSIVNADAEARYLSPGELDRIKAFVTGGAARLRIAETLTGSRETI   50
ApcA1 Synechococcus sp. PCC 7002 (WP_012307540.1)       ----------MSIVTKSIVNADAEARYLSPGELDRIKAFVTSGESRLRIAETLTGSRERI   50
ApcA1 Calothrix sp. PCC 7507 (WP_015126817.1)           ----------MSIVTKAIVNADAEARYLSPGELDRIKSFVGSGERRLRIAQVLTDNRERL   50
ApcA1 Thermosynechococcus vestitus BP-1 (WP_011056801.1) MRDVLSGVSNMSVVTKSIVNADAEARYLSPGELDRIKNFVSTGERRLRIAQTLTENRERI   60
ApcA1 Anabaena sp. PCC 7120 (WP_010994198.1)            ----------MSIVTKSIVNADAEARYLSPGELDRIKSFVAGGQQRLRIAQALTDNRERL   50
ApcA1 Chlorogloeopsis sp. PCC 9212 (WP_026087462.1)     ----------MSIVTKSIVNADAEARYLSPGELDRIKSFVSGGEKRLRIAQVLTDNRERI   50
ApcA1 Chroococcidiopsis thermalis PCC 7203 (WP_015156256.1) ----------MSIVTKSIVNADAEARYLSPGELDRIKSFVTSGERRLRIAQALTDNRERI   50
```

```
ApcA1 Fischerella thermalis PCC 7521 (WP_009457007.1)       ----------MSIVTKAIVNADAEARYLSPGELDRIKSFVTSGERRLRIAQVLTDNRERI     50
ApcA1 Leptolyngbya sp. JSC-1 (WP_036003991.1)               ----------MSIVTKSIVNADAEARYLSPGELDRIKGFVTSGERRLRIAQVLTDSRERI     50
                                                                      *::: : :  :*   *:    *:         .  :      :      :

                                                            BBBBBBBBBBBlllllllllE---EEEEEEEEEEEEEEEEEEEEEEEEllFFFFFFFF
ApcD4 Synechococcus sp. A1463 (WP_099812040.1)              VQEGSKRFWAKCPNTPSNKGNP---QKTALCQRDQGWYIRLVSYCILAGNDKPLEDIGLN    107
ApcD4 Chroococcidiopsis sp. FACHB-1243 (WP_192159074.1)     VQEGSQRFWKVIPNTPSNSGDP---KKTALCQRDQAWYLRLITYAVLAGNMKPLDDIGIN    107
ApcD4 Chroococcidiopsis thermalis PCC 7203 (WP_015156293.1) VQEGSQRFWKVIPNTPSNSGDP---KKTALCQRDQAWYLRLITYAVLAGNMKPLDDIGIN    107
ApcD4 Gloeocapsa sp. PCC 7428 (WP_015190166.1)             VQEGSRRFWQVVPNTPSNSGDP---QKTALCQRDQSWYLRLISYAVLAGNMKPLEDIGVD    107
ApcD4 Chlorogloeopsis sp. PCC 9212 (WP_016874155.1)        VEEGSRRFWKIVPNTPSNSGDP---KKTALCQRDQSWYLRLITYAVLAGNTKPLEDIGVD    107
ApcD4 Halomicronema hongdechloris (WP_080809693.1)         VDEGSKRYWAQCPDAPSNSGDP---QKTANCQRDQGWYIRIISYCVLAGNGKPMEDLGIE    107
ApcD4 Leptolyngbya sp. PCC 6406 (WP_008312402.1)          VDEGSKRFWAQCPNTPSNSGDS---QKTMLCQRDQGWYIRLVSYCVLAGNAKPLEDIGLD    107
ApcD4 Xenococcus sp. PCC 7305 (WP_006512043.1)            VEEGSKQFWAQCSNTPSNKGDA---TKTALCQRDQGWYIRLVSYCVLAGNSKPLEDIGLD    107
ApcD4 Pleurocapsa sp. CCALA 161 (WP_106232622.1)          VDQGSKQFWSQCSNTPSNSGDS---QKTALCQRDQGWYIRLVSYCVLAGNSKPLEDIGLD    107
ApcD4 Synechococcus sp. PCC 7335 (WP_006454943.1)         VDEGSKQFWNQCPNTPSNSGDK---QKTALCQRDQGWYIRLVSYCILAGNSKPLEDIGLD    107
ApcD4 Romeria gracilis (WP_193906454.1)                   VEEGSKRFWEQCPNTPSNSGDK---QKTALCQRDQGWYIRLVSYCLLAGNSKPLEDIGLD    107
ApcD4 Gloeomargarita lithophora (WP_071455395.1)          VEEGSKRFWAKCSNTPSNRGNA---QKTALCQRDQGWYIRLVSYCVLAGNDKPLDDIGLN    107
ApcD2 Synechococcus sp. PCC 7335 (WP_006455320.1)         VEKGARRFWQRCPITPSNSDNQ---QFQASCLRDQAWFIRLISYAVAVGDVDPLEASGVR    107
ApcD2 Leptolyngbya sp. JSC-1 (IMG: 2022833634)            VEQATQRFWQRCPVTPSNSGNP---TFQSSCMRDQSWYVRLVTYAIVLGDIEPIETSGVR    107
ApcD2 Chroococcidiopsis thermalis PCC 7203 (WP_015153115.1) VERGSLKFWECCPDTPSNSGNR---TYRASCLRDQDWYIRLIAYTVIVGDVEPLKDIGIV    107
ApcD2 Calothrix sp. PCC 7507 (WP_015126588.1              VEKASLKFWERCHDTPSNSGNR---IYRSSCLRDQSWYIRLITYSIVLQDIEPLAEIGTI    107
ApcD2 Chlorogloeopsis sp. PCC 9212 (WP_016873422.1)        VEKASLKFWERCPNTPSNSGNR---MYRNSCLRDQSWYIRLITYALVVGDVEPLAEIGTI    107
ApcD2 Fischerella thermalis PCC 7521 (WP_009453700.1)      VEKASLKFWERCPNTPSNSGNR---MYRNSCLRDQSWYIRLITYAVVVGDVEPLAAIGTI    107
ApcD3 Leptolyngbya sp. JSC-1 (MBF2050150.1)               VQKGSQRFWKRCPVTPSNSGNP---RKTASCQRDQGWYIRLVAYCVLAGSEQPLADIGTV    107
ApcD3 Calothrix sp. PCC 7507 (WP_015126586.1)             VEKASDKFWKVCPVTPSNSGNM---RKTASCQRDQGWYIRLVAYCILAGNEQPLEDIGTI    107
ApcD3 Chlorogloeopsis sp. PCC 9212 (WP_016873424.1)        VEKASQKFWKRCPITPSNSGNM---RKTASCQRDQGWYIRLVAYCVLAGSEQPLTEIGTV    107
ApcD3 Fischerella thermalis PCC 7521 (WP_009453698.1)      VERASQKFWKRCPVTPSNSGNM---RKTASCQRDQGWYIRLVAYCVLAGNEQPLTEIGTV    107
ApcD3 Synechococcus sp. PCC 7335 (WP_006456515.1)         VERGTRKFWQICPRTPSNSGSP---RKTEAAQRDMSWYIRLISYCLLAGNDQPLREIGLL    107
ApcD3 Chroococcidiopsis thermalis PCC 7203 (WP_015153117.1) VERATQKFWKICPRTPSNSGNA---RKTEAAMRDIGWYIRLVSYCLLAGNEKPLEEIGLI    107
ApcD5 Synechococcus sp. PCC 7335 (WP_006456289.1)         VQNGSQKFWERCPNTPSNSGVD---RKTASCQRDQGWYVRLIAYSILAGSERPLEDIGTV    107
ApcD5 Chroococcidiopsis thermalis PCC 7203 (WP_015153113.1) VQNGSARFWERVPNTPSNSGNE---RKTASCQRDQGWYIRLIAYSVLAGSEKPLEEIGTI    107
ApcD5 Leptolyngbya sp. JSC-1 (IMG: 2022833632)            VQNASAKFWERCPVTPSNSGNP---RKTASCQRDQGWYIRLVAYSVLAGSEKPLSEIGTV    107
ApcD5 Calothrix sp. PCC 7507 (WP_015126590.1)             VQNGSLKFWERCPNTPSNSGNE---RKTASCQRDQGWYVRLVAYSILAGSEKPLEEIGTI    107
ApcD5 Chlorogloeopsis sp. PCC 9212 (WP_016873420.1)        VQRGSQRFWERCPNTPSNSGNE---RKTASCQRDQGWYVRLVAYSVLAGSEKPLEEIGTV    107
ApcD5 Fischerella thermalis PCC 7521 (WP_009453702.1)      VQRGSQKFWERCPNTPSNSGNE---RKTASCQRDQGWYVRLVAYSVLAGSEKPLEEIGTV    107
ApcD1 Synechococcus sp. A1463 (WP_011431562.1)              IDQAQKQLFAKRPEYIQPGGNAYGEKRYNQCLRDYDWYLRLVTYGIIAGSKEPIESIGLI    110
ApcD1 Synechocystis sp. PCC 6803 (WP_010871516.1)         VDQAQKQLFKKHPEYRAPGGNAYGQRQYNQCLRDYGWYLRLVTYGVLAGNKEPIETTGLI    110
ApcD1 Synechococcus sp. PCC 7002 (WP_012307741.1)         VDQAQKQLFSIHPEYRTSGGNAATTKQYNQCLRDYGWYLRLVTYGILAGDKDPIERIGLI    110
ApcD1 Leptolyngbya sp. PCC 7376 (WP_015135290.1)          VDEAQKKLFAIHPEYRAAGGNASTTKRYNQCLRDYGWYLRLVTYGVLAGDKDPIENIGLI    110
ApcD1 Synechococcus sp. PCC 6301 (WP_011243585.1)         VDQASRKLWQRRPDFIAPGGNAYGQRQRAQCLRDYGWYLRLITYGVLAGDKEPIESIGLL    110
ApcD1 Thermosynechococcus sp. CL-1 (QEQ1666.1)            VEQASKQLWQKRPDFISPGGNAYGQKQRALCLRDYGWYMRLITYGILAGDKDPIERTGII    130
ApcD1 Arthrospira platensis (GCE93786.1)                  VDRASAQLWKKRPDFIAPGGNAAGQRERSLCLRDYGWYLRVITYGILSGDKDPIESIGLI    110
ApcD1 Synechococcus sp. PCC 7335 (WP_006454063.1)         VEQASGALWKLRPDFIAKGGNAYGQKQRALCLRDYGWYLRLITYSVLAGDKEPIESIGLI    110
ApcD1 Leptolyngbya sp. JSC-1 (WP_036003919.1)             VQEASKELWKKRPDFIAPGGNAYGDKQRALCLRDYGWYLRLISYGVLAGDKEPIEKIGLI    110
ApcD1 Chroococcidiopsis thermalis PCC 7203 (WP_015152396.1) VQEASKKLWQKRPDFIAPGGNAYGERQRALCLRDYGWYLRLITYGVLAGDKEPIEKIGLV    110
ApcD1 Nostoc sp. PCC 7524 (WP_015139163.1)                VQEATKQLWQKRPDFIAPGGNAYGEKQRALCIRDFGWYLRLITYGVLAGDIEPIEKIGII    110
ApcD1 Anabaena sp. PCC 7120 (WP_010997797.1)              VQEATKQLWQKRPDFIAPGGNAYGERQRALCIRDFGWYLRLITYGVLAGDIEPIEKIGII    110
ApcD1 Calothrix sp. PCC 7507 (WP_015130965.1)             VQEATKQLWQKRPDFISPGGNAYGERQRSLCIRDFGWYLRLITYGVLAGDKEPIEKIGLI    110
ApcD1 Chlorogloeopsis fritschii PCC 9212 (WP_016878526.1)  VQEATKQLWQKRPDFIAPGGNAYGERQRALCIRDFGWYLRLITYGVLAGDKEPIEKIGLV    110
ApcD1 Nostoc sp. PCC 7107 (WP_015113750.1)                VQEATKQLWQKRPDFIAPGGNAYGERQRALCIRDFGWYLRLITYGVLAGDKEPIEKIGLI    110
ApcA1 Anabaena sp. PCC 7120 (WP_010994626.1)              VKQSANQLFQKRPDIVSPGGNAYGQDMTATCLRDMDYYLRLITYSIVAGDSTPIQEIGVI    110
ApcA1 Synechococcus sp. PCC 6301 (WP_011243498.1)         VKQAGNQLFQKRPDVVSPGGNAYGEDMTATCLRDLDYYLRLVTYGVVSGDITPIEEIGIV    110
ApcA1 Synechococcus sp. PCC 7335 (WP_006456136.1)         VKTAGDQLFQKRPDVVSPGGNAYGEEMTATCLRDMDYYLRLITYGVVAGDVAPIEEIGLV    110
ApcA1 Synechocystis sp. PCC 6803 (WP_010872503.1)         VKQAGDRLFQKRPDIVSPGGNAYGEEMTATCLRDMDYYLRLVTYGVVSGDVTPIEEIGLV    110
ApcA1 Synechococcus sp. PCC 7002 (WP_012307540.1)         IKSAGDALFQKRPDVVSPGGNAYGEEMTATCLRDMDYYLRLITYGVVAGDVTPIEEIGLV    110
ApcA1 Calothrix sp. PCC 7507 (WP_015126817.1)             VKQAGEQLFQKRPDVVSPGGNAYGQELTATCLRDLDYYLRLVTYGIVAGDVTPIEEIGVI    110
ApcA1 Thermosynechococcus vestitus BP-1 (WP_011056801.1)  VKQAGDQLFQKRPDVVSPGGNAYGEEMTATCLRDLDYYLRLVTYGIVAGDVTPIEEIGLV    110
ApcA1 Anabaena sp. PCC 7120 (WP_010994198.1)              VKQAGDQLFQKRPDVVSPGGNAYGQEMTATCLRDLDYYLRLVTYGIVAGDVTPIEEIGVI    110
ApcA1 Chlorogloeopsis sp. PCC 9212 (WP_026087462.1)        VKQAGDQLFQKRPDVVSPGGNAYGQEMTATCLRDLDYYLRLVTYGVVAGDVTPIEEIGIV    110
ApcA1 Chroococcidiopsis thermalis PCC 7203 (WP_015156256.1) VKQAGDQLFQKRPDVVSPGGNAYGQEMTATCLRDLDYYLRLITYGVVAGDVTPIEEIGIV    110
ApcA1 Fischerella thermalis PCC 7521 (WP_009457007.1)      VKQAGDQLFQKRPDVVSPGGNAYGQEMTATCLRDLDYYLRLITYGVVSGDVTPIEEIGVV    110
ApcA1 Leptolyngbya sp. JSC-1 (WP_036003991.1)               VKQAGDQLFQKRPDVVSPGGNAYGEEMTATCLRDLDYYLRLITYGVVSGDVTPIEEIGIV    110
                                                            :. .    :           .           . **  :::*:::* :   .  *:   *

                                                            lFFFFFFFFll-lGGGGGGGGGGGGGGGGGllllHHHHHHHHHHHHHHHl
ApcD4 Synechococcus sp. A1463 (WP_099812040.1)              GMREMYISLGV-PLPNLRVAMSCLKEVAAGILSSEEMALAAPYFDRLIRAF---------    157
ApcD4 Chroococcidiopsis sp. FACHB-1243 (WP_192159074.1)     GMREMYVSLGV-PVSNIGSCMRSLKEVATGLMSREEADLVKPYFDYLIRAMY--------    158
ApcD4 Chroococcidiopsis thermalis PCC 7203 (WP_015156293.1) GMREMYVSLGV-PVSNIGSCMRSLKEVATGLMSREEADLVKPYFDYLIRAMY--------    158
ApcD4 Gloeocapsa sp. PCC 7428 (WP_015190166.1)             GMREMYTSLGV-PVSNIGNCMRCLKEVATNMMSSEEAAIAKPYFDYLIRAMY--------    158
ApcD4 Chlorogloeopsis sp. PCC 9212 (WP_016874155.1)        GMREMYTSLGV-PVSNIGTCMRCLKEVASGMMNREEAAIAGPYFDYLIRAMY--------    158
ApcD4 Halomicronema hongdechloris (WP_080809693.1)         GLRESYVSLGVLPLSYHKVAYRCIKEVAMEILTAEEGALVAPYFDQLIRAF---------    158
ApcD4 Leptolyngbya sp. PCC 6406 (WP_008312402.1)          GMRDMYLSLGV-PLSNLKLAMKCLKDVAIGLLTAEEGALAAPYFEQLIRAF---------    157
ApcD4 Xenococcus sp. PCC 7305 (WP_006512043.1)            GMRDMYVSLQV-PLANLKIAMRCIKSVATGLLNSEEAALAAPYFDELIRAF---------    157
ApcD4 Pleurocapsa sp. CCALA 161 (WP_106232622.1)          GMRDMYTSLQV-PLANLKLAMRCLKSVAMGLLTSEEAALAGPYFDQLIRAF---------    157
ApcD4 Synechococcus sp. PCC 7335 (WP_006454943.1)         GMRDMYVSLNV-PLTNLKTAMRCIKQSAMGVLSSEEASLAGPYFDQLIRAF---------    157
ApcD4 Romeria gracilis (WP_193906454.1)                   GMRDMYVSLNV-PLANLKLAMRCLKQVATGLLSSEEAALASPYFDQLIRAF---------    157
ApcD4 Gloeomargarita lithophora (WP_071455395.1)          GMREMYVSLGV-PLANLRMAMGSLKDVAAGLMSGEEMALAAPYFDRLIRAF---------    157
ApcD2 Synechococcus sp. PCC 7335 (WP_006455320.1)         GVREMYLSLEV-PLRSVALCMRSLKEVTLAMLSREDAAEVGPYFDYLIAGLMP-------    159
ApcD2 Leptolyngbya sp. JSC-1 (IMG: 2022833634)            GAKEMYASLGV-PLTNLVECMRCLKEVALELLALDDAVEVAPYFDYLIQGLKP-------    159
ApcD2 Chroococcidiopsis thermalis PCC 7203 (WP_015153115.1) GVKEMYESLEI-PLRNWVECIRCLKEVTLDLLSREDAAEVTPYFDCLIQGMIP-------    159
ApcD2 Calothrix sp. PCC 7507 (WP_015126588.1              GVQEMYNSLEI-PLANLVIAIRCLKEVSLDLFNLEDAAELAPYFDYLIQELTP-------    159
ApcD2 Chlorogloeopsis sp. PCC 9212 (WP_016873422.1)        GVKEMYDSLEI-PLPNLVEAIRCLKEVSLDLFTLEDAAEVAPYFDYLIQSLMP-------    159
ApcD2 Fischerella thermalis PCC 7521 (WP_009453700.1)      GVKEMYESLEI-PLPNLVEAIRCLKEVSLDLFTLEDATEIAPYFDYLIQSLMP-------    159
ApcD3 Leptolyngbya sp. JSC-1 (MBF2050150.1)               GMKEMYISLGI-PLANWVEAVQCLKEEAIALLGQPDAAVVAPYFDHIIQTLALPGTPYFV    166
ApcD3 Calothrix sp. PCC 7507 (WP_015126586.1)             GMKEMYASLGI-PIFNWVEAVRCIKEAAREFLGEEDAAIAIPYFDHIIQTLASPGSPYFM    166
ApcD3 Chlorogloeopsis sp. PCC 9212 (WP_016873424.1)        GMQQMYESLGI-PLSNWVEAVRCIKEEAQALLGDEDAAEVTPYFDHIIQTLSFPGAPYFM    166
ApcD3 Fischerella thermalis PCC 7521 (WP_009453698.1)      GMQQMYQSLGI-PLSNWVEAVRCIKEEAQALLGDEDAAEVTPYFDYIIQALSFPGAPYFM    166
ApcD3 Synechococcus sp. PCC 7335 (WP_006456515.1)         GMKELYTNIGI-PLDNILQYLRCLKAEAIALLSEAEAEAIIPYFDQIIQELVRPGPSYFG    166
ApcD3 Chroococcidiopsis thermalis PCC 7203 (WP_015153117.1) GMKELYNSVGI-PLENVRQYMLCVKAEVSAMLTPEDAAEVIPYFDLILQVISSPGAPYFQ    166
ApcD5 Synechococcus sp. PCC 7335 (WP_006456289.1)         GIKEMYNNLEI-PIRNIAECMRCLKEEAMAVLSDEDAQEVAAYFDLIIQSLS--------    158
ApcD5 Chroococcidiopsis thermalis PCC 7203 (WP_015153113.1) GIKEMYNNLEI-PLRNIVECMRCLKEEALSLMSEEDALEVSAYFDYVMRSLS--------    158
ApcD5 Leptolyngbya sp. JSC-1 (IMG: 2022833632)            GIKEMYNSLEI-PLKNLVEAMRCVKDEAISLMSEDDAVEVAPYFDYIIRALS--------    158
ApcD5 Calothrix sp. PCC 7507 (WP_015126590.1)             GIKEMYNNLEI-PLRNIVEAMRCVKEEAISLMSEEDAVEVGPYFDYIIRALS--------    158
ApcD5 Chlorogloeopsis sp. PCC 9212 (WP_016873420.1)        GIKEMYNNLEI-PLRNIVEAMRCIKEEAVSMMSEEDAVEVGPYFDYIIRALS--------    158
ApcD5 Fischerella thermalis PCC 7521 (WP_009453702.1)      GIKEMYNNLEI-PLRNIVEAMRCIKEEAVSMMSEEDAVEVGPYFDYIIRALS--------    158
ApcD1 Synechococcus sp. A1463 (WP_011431562.1)              GVREMYNALNV-PIAGMIDAIVFLKEAALSLLDPDSAAEAAPYFDYIINAMS--------    161
ApcD1 Synechocystis sp. PCC 6803 (WP_010871516.1)         GVKEMYNSLNV-PVPGMVDAVTVLKDAALGLLSAEDANETAPYFDYIIQFMS--------    161
ApcD1 Synechococcus sp. PCC 7002 (WP_012307741.1)         GVKEMYNALGV-PVPGMVDAIRCLKDAALGVLDSEEARIAAPYFDFITQAMS--------    161
ApcD1 Leptolyngbya sp. PCC 7376 (WP_015135290.1)          GVKEMYNALDV-PVTGMIDAIRCLKEAALGVLDLEEASIAAPYFDFITQSMS--------    161
ApcD1 Synechococcus sp. PCC 6301 (WP_011243585.1)         GAREMYNSLGV-PLPGMAEAIRTLKEASLALLSSADATVAAPYFDFLIQGMETI------    163
ApcD1 Thermosynechococcus sp. CL-1 (QEQ1666.1)            GVREMYNSLGV-PMTGMAEAMRCLKDASLALLSTEDAEVAAPYFDYIIQEMS--------    171
ApcD1 Arthrospira platensis (GCE93786.1)                  GVKEMYNSLGV-PMPGMVEAIRCLKEASLALLDDEDAKEAAPYFDFIIQAMS--------    161
ApcD1 Synechococcus sp. PCC 7335 (WP_006454063.1)         GVREMYNALDV-PVPGMVEAIRCLKNASLSLMNEEDAAEATPYFDYIIQAMSA-------    162
ApcD1 Leptolyngbya sp. JSC-1 (WP_036003919.1)             GVKEMYNSLGV-PVPGMVESIRCLKNAALALMSDEDATEAAPYFDYIIQTMS--------    161
```

```
ApcD1 Chroococcidiopsis thermalis PCC 7203 (WP_015152396.1) GVREMYNSLGV-PVPGMVESIRCLKNASLSLLSAEEAAEAAPYFDYIIQAMS--------     161
ApcD1 Nostoc sp. PCC 7524 (WP_015139163.1)                  GVREMYNSLGV-PVPGMVEAINALKKASLDLLSAEDAAEAAPYFDYIIQAMS--------     161
ApcD1 Anabaena sp. PCC 7120 (WP_010997797.1)                GVREMYNSLGV-PVPGMVEAINSLKKASLDLLSSEDAAAAAPYFDYIIQAMS--------     161
ApcD1 Calothrix sp. PCC 7507 (WP_015130965.1)               GVREMYNSLGV-PVPGMVEAINSLKKASLDLLSTEDAAETAPYFDYIIQAMS--------     161
ApcD1 Chlorogloeopsis fritschii PCC 9212 (WP_016878526.1)   GVREMYNSLGV-PVPGMVEAINCLKKASLDLLNAEDAAEAAPYFDYIIQAMS--------     161
ApcD1 Nostoc sp. PCC 7107 (WP_015113750.1)                  GVREMYNSLGV-PVPGMVEAINSLKKASLDLLSAEDAAAASPYFDYIIQAMS--------     161
ApcA1 Anabaena sp. PCC 7120 (WP_010994626.1)                GVREMYRSLGT-PIEAVAESIRAMKYVATSMMSVEDRAEVDTYFDYLIGAMQ--------     161
ApcA1 Synechococcus sp. PCC 6301 (WP_011243498.1)          GVREMYKSLGT-PIEAVAEGVRELKSAATALLTGEDADEAGAYFDYVIGALS--------     161
ApcA1 Synechococcus sp. PCC 7335 (WP_006456136.1)          GAREMYNSLGT-SIPAMADSIRCMKSVAGSMMSGDDALEAASYFDYVIGGLQ--------     161
ApcA1 Synechocystis sp. PCC 6803 (WP_010872503.1)          GVREMYRSLGT-PIEAVAQSVREMKEVASGLMSSDDAAEASAYFDFVIGKMS--------     161
ApcA1 Synechococcus sp. PCC 7002 (WP_012307540.1)          GVREMYKSLGT-PVDAVAQAVREMKAVATGMMSGDDAAEAGAYFDYVIGAME--------     161
ApcA1 Calothrix sp. PCC 7507 (WP_015126817.1)               GARELYKSLGT-PIEGVAEGIRGLKNVAASLLSAEDASEAGSYFDYLVGALL--------     161
ApcA1 Thermosynechococcus vestitus BP-1 (WP_011056801.1)    GVREMYNSLGT-PIPAVAEGIRAMKNVACSLLSAEDAAEAGSYFDFVIGAMQ--------     161
ApcA1 Anabaena sp. PCC 7120 (WP_010994198.1)                GVREMYKSLGT-PIEAVGEGVRALKNAASTLLSAEDAAEAGSYFDYVVGALQ--------     161
ApcA1 Chlorogloeopsis sp. PCC 9212 (WP_026087462.1)        GVREMYKSLGT-PIDAVAAGVSAMKNVAASLLSADDASEAGAYFDYVAGALA--------     161
ApcA1 Chroococcidiopsis thermalis PCC 7203 (WP_015156256.1) GVREMYKSLGT-PIEAVAEGVRAMKNVATSMMSGEDAGEAGSYFDYLVGAMQ--------     161
ApcA1 Fischerella thermalis PCC 7521 (WP_009457007.1)       GVREMYKSLGT-PIDAVAAGVNAMKNVAASLLSGEDAAEAGAYFDYLVGAMS--------     161
ApcA1 Leptolyngbya sp. JSC-1 (WP_036003991.1)              GVREMYKSLGT-PIDAVAEGVRAMKSVATSLLSGDDAAEAGTYFDYVIGAMQ--------     161
                                                            * :: *  :    :         :*     .:   .      **: :   :

ApcD4 Synechococcus sp. A1463 (WP_099812040.1)             ---------------        157
ApcD4 Chroococcidiopsis sp. FACHB-1243 (WP_192159074.1)    ---------------        158
ApcD4 Chroococcidiopsis thermalis PCC 7203 (WP_015156293.1) ---------------        158
ApcD4 Gloeocapsa sp. PCC 7428 (WP_015190166.1)             ---------------        158
ApcD4 Chlorogloeopsis sp. PCC 9212 (WP_016874155.1)        ---------------        158
ApcD4 Halomicronema hongdechloris (WP_080809693.1)         ---------------        158
ApcD4 Leptolyngbya sp. PCC 6406 (WP_008312402.1)           ---------------        157
ApcD4 Xenococcus sp. PCC 7305 (WP_006512043.1)             ---------------        157
ApcD4 Pleurocapsa sp. CCALA 161 (WP_106232622.1)           ---------------        157
ApcD4 Synechococcus sp. PCC 7335 (WP_006454943.1)          ---------------        157
ApcD4 Romeria gracilis (WP_193906454.1)                    ---------------        157
ApcD4 Gloeomargarita lithophora (WP_071455395.1)           ---------------        157
ApcD2 Synechococcus sp. PCC 7335 (WP_006455320.1)          ---------------        159
ApcD2 Leptolyngbya sp. JSC-1 (IMG: 2022833634)             ---------------        159
ApcD2 Chroococcidiopsis thermalis PCC 7203 (WP_015153115.1) ---------------        159
ApcD2 Calothrix sp. PCC 7507 (WP_015126588.1               ---------------        159
ApcD2 Chlorogloeopsis sp. PCC 9212 (WP_016873422.1)        ---------------        159
ApcD2 Fischerella thermalis PCC 7521 (WP_009453700.1)      ---------------        159
ApcD3 Leptolyngbya sp. JSC-1 (MBF2050150.1)                NDGTSEY--------        173
ApcD3 Calothrix sp. PCC 7507 (WP_015126586.1)              NDGRTDW--------        173
ApcD3 Chlorogloeopsis sp. PCC 9212 (WP_016873424.1)        NDGRLDW--------        173
ApcD3 Fischerella thermalis PCC 7521 (WP_009453698.1)      NDGRSDW--------        173
ApcD3 Synechococcus sp. PCC 7335 (WP_006456515.1)          IKDRSARQSARQAAA        181
ApcD3 Chroococcidiopsis thermalis PCC 7203 (WP_015153117.1) NNGRTDWQR------        175
ApcD5 Synechococcus sp. PCC 7335 (WP_006456289.1)          ---------------        158
ApcD5 Chroococcidiopsis thermalis PCC 7203 (WP_015153113.1) ---------------        158
ApcD5 Leptolyngbya sp. JSC-1 (IMG: 2022833632)             ---------------        158
ApcD5 Calothrix sp. PCC 7507 (WP_015126590.1)              ---------------        158
ApcD5 Chlorogloeopsis sp. PCC 9212 (WP_016873420.1)        ---------------        158
ApcD5 Fischerella thermalis PCC 7521 (WP_009453702.1)      ---------------        158
ApcD1 Synechococcus sp. A1463 (WP_011431562.1)             ---------------        161
ApcD1 Synechocystis sp. PCC 6803 (WP_010871516.1)          ---------------        161
ApcD1 Synechococcus sp. PCC 7002 (WP_012307741.1)          ---------------        161
ApcD1 Leptolyngbya sp. PCC 7376 (WP_015135290.1)           ---------------        161
ApcD1 Synechococcus sp. PCC 6301 (WP_011243585.1)          ---------------        163
ApcD1 Thermosynechococcus sp. CL-1 (QEQ1666.1)             ---------------        171
ApcD1 Arthrospira platensis NIES46 (GCE93786.1)            ---------------        161
ApcD1 Synechococcus sp. PCC 7335 (WP_006454063.1)          ---------------        162
ApcD1 Leptolyngbya sp. JSC-1 (WP_036003919.1)              ---------------        161
ApcD1 Chroococcidiopsis thermalis PCC 7203 (WP_015152396.1) ---------------        161
ApcD1 Nostoc sp. PCC 7524 (WP_015139163.1)                 ---------------        161
ApcD1 Anabaena sp. PCC 7120 (WP_010997797.1)               ---------------        161
ApcD1 Calothrix sp. PCC 7507 (WP_015130965.1)              ---------------        161
ApcD1 Chlorogloeopsis fritschii PCC 9212 (WP_016878526.1)  ---------------        161
ApcD1 Nostoc sp. PCC 7107 (WP_015113750.1)                 ---------------        161
ApcA1 Anabaena sp. PCC 7120 (WP_010994626.1)               ---------------        161
ApcA1 Synechococcus sp. PCC 6301 (WP_011243498.1)          ---------------        161
ApcA1 Synechococcus sp. PCC 7335 (WP_006456136.1)          ---------------        161
ApcA1 Synechocystis sp. PCC 6803 (WP_010872503.1)          ---------------        161
ApcA1 Synechococcus sp. PCC 7002 (WP_012307540.1)          ---------------        161
ApcA1 Calothrix sp. PCC 7507 (WP_015126817.1)              ---------------        161
ApcA1 Thermosynechococcus vestitus BP-1 (WP_011056801.1)   ---------------        171
ApcA1 Anabaena sp. PCC 7120 (WP_010994198.1)               ---------------        161
ApcA1 Chlorogloeopsis sp. PCC 9212 (WP_026087462.1)        ---------------        161
ApcA1 Chroococcidiopsis thermalis PCC 7203 (WP_015156256.1) ---------------        161
ApcA1 Fischerella thermalis PCC 7521 (WP_009457007.1)      ---------------        161
ApcA1 Leptolyngbya sp. JSC-1 (WP_036003991.1)              ---------------        161
```

B

β-subunits (B3, B2, and B1)

```
                                                             lllXXXXXXXXXXXXlllllYYYYYYYYYYYYlAAAAAAAAAAAAlBBBBBBBBBBB
ApcB3 Synechococcus sp. A1463 (WP_099812041.1)             -MKDTITSLINPADEKGSYLDAAALEQLNRYFQSGNMRVKAAKTISSSASSIISKTVAKS     59
ApcB3 Chlorogloeopsis sp. PCC 9212 (WP_016874154.1)        -MQDTITSLINPADLRGKYLDNTELDKLRKYFQSGELRVKAAATISENAANIVSQAVANS     59
ApcB3 Chroococcidiopsis thermalis PCC 7203 (WP_015156292.1) -MQDTITSVINPADRQGKYLDTPELEKLRKYFQTGELRVKAAATISENASSIVSQAVANS     59
ApcB3 Synechococcus sp. PCC 7335 (WP_006453385.1)          -MQDTITSLINPADEKGQYLEGGDLDSLKQYLQSGATRVKAAGQIGDSAASIISKTVERS     59
ApcB3 Gloeocapsa sp. PCC 7428 (WP_015190165.1)             -MQDTITSLINPADQRGKYLETEELEKLRRYFQSGELRVKAASAISNNAANIIREAVANS     59
ApcB3 Gloeomargarita lithophora D10 (WP_071455396.1)       -MQDTITSLINPADEQGQYLNAAALDQLNKYFQKGAVRVQAASTISDTASSIISKTVAKS     59
ApcB3 Halomicronema hongdechloris (WP_080809690.1)         -MQDIITAAINPADERCAYLEDSSLEKLRQYYQSGTLRLKAATQIGNSAASIISDAVRKS     59
ApcB3 Xenococcus sp. PCC 7305 (WP_006512042.1)             -MQDTITSLINPADEKGQYLEGQELDQLKKYFQSGSLRVKAADQIGSAAASIITESVAKS     59
ApcB3 Leptolyngbya sp. PCC 6406 (WP_008312401.1)           -MQDTITATINPADEQGIYLEGEQLDALKAYFQSGTLRVKAASQIGDSAASIISETVAKS     59
ApcB2 Synechococcus sp. PCC 7335 (WP_006454442.1)          -MQDAITTLINTSDAQGKYLDDSSLDTLQEYFRSGDLRAKAAMTISANASTIVTKTVAKS     59
ApcB2 Leptolyngbya sp. JSC-1 (WP_036011375.1)              -MQDAITALINSSDVQGRYLDNNGLDKLRSYFQSGEMRARAAITISANASSLVTQAVAKS     59
ApcB2 Chlorogloeopsis sp. PCC_9212 (WP_016873421.1)        -MQDAITSLINSSDVQGKYLDNNSLDKLQHYYHTGDMRARAATAISANAKTIVTQTVAKS     59
ApcB2 Fischerella thermalis PCC 7521 (WP_009453701.1)      -MQDAITSLINSSDVQGKYLDNNSLEKLQHYYHTGDMRARAATTISANAKTIVTQTVAKS     59
ApcB2 Chroococcidiopsis thermalis PCC 7203 (WP_015153114.1) -MQDAITALINSSDVQGRYLDPSSLDKLQNYFQSGDMRAKTAIAVSANAKNIVTKTVAKS     59
ApcB2 Calothrix sp. PCC 7507 (WP_015126589.1)              -MQDAITALINSSDVQGKYLDSSSLEKLQNYFHSGDVRARAATTVSANAKNIVTKAVAKS     59
ApcB1 Thermosynechococcus vestitus BP-1 (WP_011056800.1)   -MQDAITAVINASDVQGKYLDTAAMEKLKAYFATGELRVRAASVISANAANIVKEAVAKS     59
ApcB1 Calothrix sp. PCC 7507 (WP_015126816.1)              MAQDAITAVINSADVQGKYLDTAAIEKLKGYFSSGDLRVRAAGTISANAAVIVKEAVAKS     60
ApcB1 Chroococcidiopsis thermalis PCC 7203 (WP_015156257.1) -MQDAITSVINTSDVQGKYLDTAAMEKLKGYFQSGELRVRAATTIAANAAAIVKEAVAKS     59
ApcB1 Anabaena sp. PCC 7120 (WP_010994199.1)               MAQDAITAVINSADVQGKYLDTAALEKLKAYFSTGELRVRAATTISANAAAIVKEAVAKS     60
```

```
ApcB1 Chlorogloeopsis sp. PCC 9212 (WP_016874648.1)         -MQDAITAVINSSDVQGKYLDTAALEKLKGYFSTGELRVRAATTIAANAAAIVKEAVAKS     59
ApcB1 Fischerella thermalis PCC 7521 (WP_009457009.1)       -MQDAITAVINSSDVQGKYLDTAALEKLKSYFSTGELRVRAATTIAANAAAIVKEAVAKS     59
ApcB1 Synechococcus sp. PCC 6301 (WP_011243499.1)           -MQDAITAVINASDVQGKYLDSSALDRLKSYFQSGELRVRAAATISANSALIVKEAVAKS     59
ApcB1 Synechococcus sp. PCC 7002 (WP_012307539.1)           -MQDAITSVINSADVQGKYLDGSAMDKLKAYFTTGALRVRAASTISANAAAIVKEAVAKS     59
ApcB1 Synechococcus sp. PCC 7335 (WP_006456852.1)           -MQDAITAVINASDVQGKYLDSSSMDKLKAYFQTGELRVRAATSISANAAEIVKEAVAKS     59
ApcB1 Synechocystis sp. PCC 6803 (WP_010872504.1)           -MQDAITAVINSADVQGKYLDGAAMDKLKSYFASGELRVRAASVISANAATIVKEAVAKS     59
ApcB1 Leptolyngbya sp. JSC-1 (MBF2047188.1)                 -MQDAITSVINSADVQGKYLDSTAMDKLKSYFSSGELRVRAATAISANAAAIVKEAVAKS     59
                                                              :* **: ** :* :  **:   :: *. *  .*  * ::*  :.  :  :: .:* .*

                                                            llllllllllllllEEEEEEEEEEEEEEEEEEEEEEEEllllllFFFFlllllFFFFFFFF
ApcB3 Synechococcus sp. A1463 (WP_099812041.1)              LLYGDITLPGGNMYPTRRYAACLRDLTYFLRYATYAMLAADPSILDERVLQGLKETYITL     119
ApcB3 Chlorogloeopsis sp. PCC 9212 (WP_016874154.1)        LLYGDITCPGGNMYPTRRYAACLRDLTLFLRYATYAMLADDASVLDERVLDGLKETYNSL     119
ApcB3 Chroococcidiopsis thermalis PCC 7203 (WP_015156292.1) LLYGDITCPGGNMYPTRRYAACIRDLTLFLRYATYAMLADDPSIIEERVLFGLKETFSTL     119
ApcB3 Synechococcus sp. PCC 7335 (WP_006453385.1)           LLYGDITLPGGNMYPTRRYAACLQDLTYFLRYATYAMLADDASIIDERILNGLKDTYSSL     119
ApcB3 Gloeocapsa sp. PCC 7428 (WP_015190165.1)              LLYGDITCPGGNMYPTRRYAACIRDLTLFLRYATYAMLAADPSILDERVLDGLKETYNSL     119
ApcB3 Gloeomargarita lithophora D10 (WP_071455396.1)        LLYGDITLPGGNMYPTRRYAACLRDLNYFLRYATYAMLAADASILDERVLNGLKETYAAL     119
ApcB3 Halomicronema hongdechloris (WP_080809690.1)          LLYGTITEPGGNMYPWRRYAACLRDLNYFLRYATYAMLAADASIIDERVLNGLRETYLSL     119
ApcB3 Xenococcus sp. PCC 7305 (WP_006512042.1)              LLYGDITLPGGNMYPTRRYAACLRDLTYFLRYAVYAMLADDPSILDERVLNGLKDTYLSL     119
ApcB3 Leptolyngbya sp. PCC 6406 (WP_008312401.1)            LLYGDITCPGGNMYPTRRYAACLRDLTYFLRYATYAMLAADASILDERVLNGLKETYSSL     119
ApcB2 Synechococcus sp. PCC 7335 (WP_006454442.1)           LLYTDITGPGGNMYTCRRYAACIRDMDFFLRYGTYAMLAGDASILDERVLNGLKETYNSL     119
ApcB2 Leptolyngbya sp. JSC-1 (WP_036011375.1)               LMYTDITAPGGNMYTCRRYAACIRDLDYFLRYATYAMLAGDPSILDERILNGLRETYNSL     119
ApcB2 Chlorogloeopsis sp. PCC_9212 (WP_016873421.1)         LLYTDITAPGGNMYTCRRYAACVRDLDYFLRYATYAMLAGDPSILDERILNGLRETYNSL     119
ApcB2 Fischerella thermalis PCC 7521 (WP_009453701.1)       LLYTDITAPGGNMYTCRRYAACVRDLDYFLRYATYAMLAGDPSILDERILNGLRETYNSL     119
ApcB2 Chroococcidiopsis thermalis PCC 7203 (WP_015153114.1) LLYTDITAPGGNMYTCRRYAACVRDLDYFLRYATYAMLAGDTSILDERILNGLRETYNSL     119
ApcB2 Calothrix sp. PCC 7507 (WP_015126589.1)               LLYTDITGPGGNMYTCRRYAACIRDLDYFLRYATYAMLAGDPSILDERILNGLRETYNSL     119
ApcB1 Thermosynechococcus vestitus BP-1 (WP_011056800.1)    LLYSDITRPGGNMYTTRRYAACIRDLDYYLRYATYAMLAGDPSILDERVLNGLKETYNSL     119
ApcB1 Calothrix sp. PCC 7507 (WP_015126816.1)               LLYSDITRPGGNMYTTRRYAACIRDLDYYLRYATYAMLAGDASILDERVLNGLKETYNSL     120
ApcB1 Chroococcidiopsis thermalis PCC 7203 (WP_015156257.1) LLYSDITRPGGNMYTTRRYAACIRDLDYYLRYSTYAMLAGDPSILDERVLNGLKETYNSL     119
ApcB1 Anabaena sp. PCC 7120 (WP_010994199.1)                LLYSDITRPGGNMYTTRRYAACIRDLDYYLRYATYAMLAGDPSILDERVLNGLKETYNSL     120
ApcB1 Chlorogloeopsis sp. PCC 9212 (WP_016874648.1)         LLYSDITRPGGNMYTTRRYAACIRDLDYYLRYATYAMLAGDPSILDERVLNGLKETYNSL     119
ApcB1 Fischerella thermalis PCC 7521 (WP_009457009.1)       LLYSDITRPGGNMYTTRRYAACIRDLDYYLRYATYAMLAGDPSILDERVLNGLKETYNSL     119
ApcB1 Synechococcus sp. PCC 6301 (WP_011243499.1)           LLYSDITRPGGNMYTTRRYAACIRDLEYYLRYATYAMLAGDTSILDERVLNGLKETYNSL     119
ApcB1 Synechococcus sp. PCC 7002 (WP_012307539.1)           LLYSDVTRPGGNMYTTRRYAACIRDLDYYLRYATYAMLAGDPSILDERVLNGLKETYNSL     119
ApcB1 Synechococcus sp. PCC 7335 (WP_006456852.1)           LLYSDITRPGGNMYTTRRYAACIRDLDYYLRYSTYAMLAGDPSILDERVLNGLKETYNSL     119
ApcB1 Synechocystis sp. PCC 6803 (WP_010872504.1)           LLYSDVTRPGGNMYTTRRYAACIRDLDYYLRYATYAMLAGDASILDERVLNGLKETYNSL     119
ApcB1 Leptolyngbya sp. JSC-1 (MBF2047188.1)                 LLYSDITRPGGNMYTTRRYAACIRDLDYYLRYATYAMLAGDPSILDERVLNGLKETYNSL     119
                                                            *:*  :* ******  ******::*:  :***..***** * *:::**:* **::*: :*

                                                            lllGGGGGGGGGGGGGGGGllllHHHHHHHHHHHHHHHHHll
ApcB3 Synechococcus sp. A1463 (WP_099812041.1)              GVPIDRVIQALNAMKEVLTESLDTEASQEMAVYLDHIIAGLS-       161
ApcB3 Chlorogloeopsis sp. PCC 9212 (WP_016874154.1)        GVPVDRTIQAVQAMKEVITRQVGAEAGEQVGRHLDHICNGLS-       161
ApcB3 Chroococcidiopsis thermalis PCC 7203 (WP_015156292.1) GVPIQPTVQAIQALKEVTTRLVGAEAGQEVGTHLDHICSGLSQ       162
ApcB3 Synechococcus sp. PCC 7335 (WP_006453385.1)           GVPVEPTIQAIEAMKDVVSERVGTEAGQEVGKYLDHIIAGLR-       161
ApcB3 Gloeocapsa sp. PCC 7428 (WP_015190165.1)              GVPIQPTIQAIQAMKEVTTRLVGAEAGGEIGMYFDHICNGLS-       161
ApcB3 Gloeomargarita lithophora D10 (WP_071455396.1)        GVPIDRVVEALNAMKEVLHGAVGAEAGQELGVYLDHITAGLA-       161
ApcB3 Halomicronema hongdechloris (WP_080809690.1)          GVPIEPTIQAIQAMKEVVTQRVGADAGQEMDVYLDHIISGLS-       161
ApcB3 Xenococcus sp. PCC 7305 (WP_006512042.1)              GVPIEPTIQAVQAMKEVVTQRVGAEAGQEMDVYLDHIIAGLG-       161
ApcB3 Leptolyngbya sp. PCC 6406 (WP_008312401.1)            GVPVEATIQAVQAMKEVVTHRVGADAGQEMDVYLDHIIAGLS-       161
ApcB2 Synechococcus sp. PCC 7335 (WP_006454442.1)           GVPVGATIRAVQAMKEVVNDMLGAEAGKEVGYYFDHICSGLS-       161
ApcB2 Leptolyngbya sp. JSC-1 (WP_036011375.1)               GVPIGATIRSVQAMKEATTDLVGAEAGKEMGVYFDYICAGLS-       161
ApcB2 Chlorogloeopsis sp. PCC_9212 (WP_016873421.1)         GVPIGATIRAVQAMKEVTNSIVGAEAGKEMGVYFDYIASGLS-       161
ApcB2 Fischerella thermalis PCC 7521 (WP_009453701.1)       GVPIGATIRAVQAMKEVTNSMIGADAGKEMGVYFDYIASGLS-       161
ApcB2 Chroococcidiopsis thermalis PCC 7203 (WP_015153114.1) GVPIGATIRSVQAMKEVVTSLVGADAGREMGVYFDHIAAGLS-       161
ApcB2 Calothrix sp. PCC 7507 (WP_015126589.1)               GVPVGATIRSVQALKEVSTSIVGADAGKELGVYFDYIASGLS-       161
ApcB1 Thermosynechococcus vestitus BP-1 (WP_011056800.1)    GVPIAATVQAIQAMKEVTASLVGADAGKEMGIYFDYICSGLS-       161
ApcB1 Calothrix sp. PCC 7507 (WP_015126816.1)               GVPVGATVQAIQAIKEVTAGLVGSDAGREIGVYLDYISSGLS-       162
ApcB1 Chroococcidiopsis thermalis PCC 7203 (WP_015156257.1) GVPVGATVQAIQAMKEVTASLTGPDAGKEMGVYFDYICSGLS-       161
ApcB1 Anabaena sp. PCC 7120 (WP_010994199.1)                GVPVGATVQAIQAIKEVTASLVGADAGKEMGIYLDYISSGLS-       162
ApcB1 Chlorogloeopsis sp. PCC 9212 (WP_016874648.1)         GVPIGATVQAIQAMKEVTASLVGPDAGKEMGVYFDYISSGLS-       161
ApcB1 Fischerella thermalis PCC 7521 (WP_009457009.1)       GVPIGATVQAIQAMKEVTASLVGPDAGKEMGVYLDYICSGLS-       161
ApcB1 Synechococcus sp. PCC 6301 (WP_011243499.1)           GVPIGATVQAIQAIKEVTASLVGPDAGREMGVYLDYISSGLS-       161
ApcB1 Synechococcus sp. PCC 7002 (WP_012307539.1)           GVPVGSTVQAIQAMKEVTAGLVGADAGREMGVYFDYICSGLS-       161
ApcB1 Synechococcus sp. PCC 7335 (WP_006456852.1)           GVPVGATVQAIQAIKEVTASLVGADAGKEMGVYLDYICSGLS-       161
ApcB1 Synechocystis sp. PCC 6803 (WP_010872504.1)           GVPISSTVQAIQAIKEVTASLVGADAGKEMGVYLDYICSGLS-       161
ApcB1 Leptolyngbya sp. JSC-1 (MBF2047188.1)                 GVPIAATVQAIQAIKEVTASLVGADAGKEMGVYLDYICSGLS-       161
                                                            ***:  .:.:::*:*:.     . :*. ::  ::*:*  **
```

C

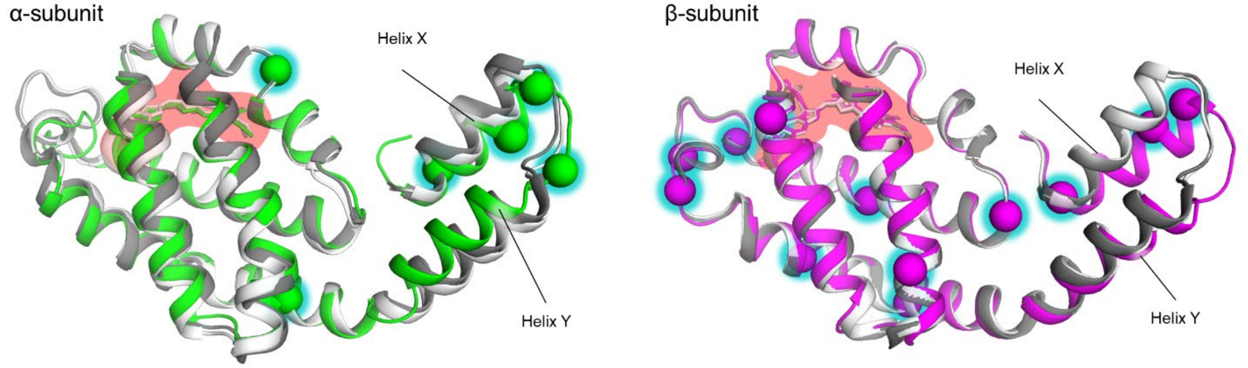

Fig. S8. Sequence alignments of α- and β-subunits for AP family members. All sequence alignments were performed using Clustal Omega (*50*). (**A**) Sequence alignments for various α-subunits. (**B**) Sequence alignments for various β-subunits. For (A) and (B), sequences are shown and colored by their gene product name (e.g., ApcD4 is shown in red font). Sequences corresponding to the FRL-AP structure (i.e., ApcD4 and ApcB3 from *Synechococcus* sp. A1463)

are shown as the top sequence. Residues in ApcD4 or ApcB3 that appear distinct from other AP sequences are highlighted in cyan. Residues that appear distinct to all FRL-AP sequences are highlighted yellow in the ApcD4 or ApcB3 sequence. Above the alignments shown in (A) and (B), letters correspond to either looping regions, labeled "*l*", or helical regions according to the nomenclature shown in **fig. S7** (e.g., "A" = helix A). Below the alignments, the Clustal Omega sequence conservation identifiers are shown. (**C**) ApcD4- and ApcB3-specific residues based on sequence alignments. The α- and β-subunits of FRL-AP are shown with the corresponding subunits from the structures of AP and AP-B. The PCB in shown with a red background. Residue positions that appear specific to ApcD4 sequences and ApcB3 sequences are shown as spheres and highlighted in cyan. Note that these correspond to the cyan highlights in panels (A) and (B).

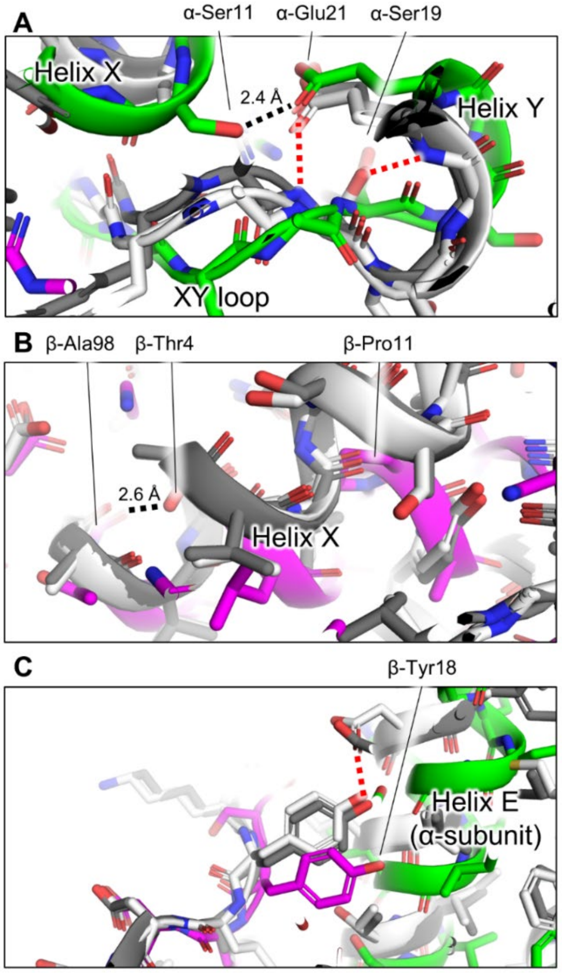

Fig. S9. Specific interactions that are implicated in FRL-AP helical nanotube formation. (**A**) The α-subunits of the FRL-AP, AP-B, and AP structures are superimposed using all atoms. The Ser-Glu H-bond important for XY-domain positioning is shown with a black dashed line. H-bonding interactions present in the AP-B and AP structures that are lacking in the FRL-AP structure are shown with red dashed lines. (**B**) The β-subunits of the FRL-AP, AP-B, and AP structures are superimposed using all atoms. The Thr-Ala H-bond important for XY-domain positioning is shown with a black dashed line. The Pro residue that causes a bend in helix X in the FRL-AP structure relative to the AP-B and AP structures is additionally labeled. (**C**) The β-subunits of the FRL-AP, AP-B, and AP structures are superimposed using atoms in the XY domain only, which allows for the visualization of the β-Tyr18 orientations differences. The H-bonding interaction found in the AP and AP-B structures is shown with a red dashed line.

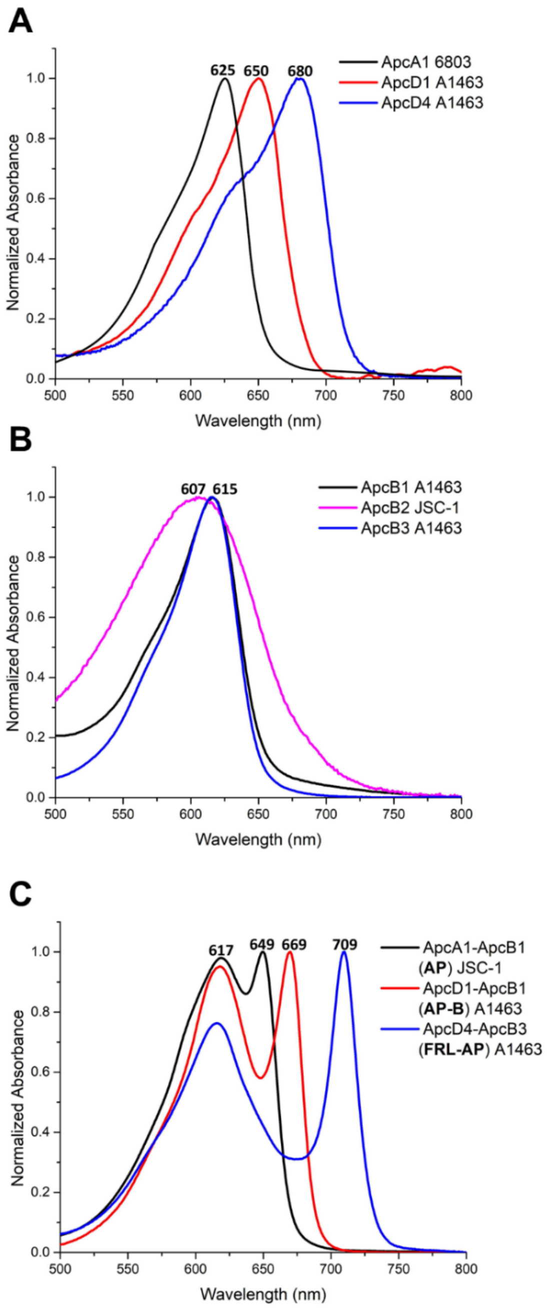

Fig. S10. Absorbance spectra of AP, AP-B, and FRL-AP and their α- and β-subunits. (**A**) Absorbance spectra for ApcA1 from *Synechocystis* sp. PCC 6803 (black line) and ApcD1 (red line) and ApcD4 (blue line) from the thermophilic cyanobacterium *Synechococcus* sp. A1463. (**B**) Absorbance spectra for representative AP beta subunits, including ApcB1 (black line) and ApcB3 (blue line) from the thermophilic cyanobacterium *Synechococcus* sp. A1463 and ApcB2 (magenta line) from *Leptolyngbya* sp. JSC-1. (**C**) Repeat of the absorbance spectra from **Fig. 5B** in the main text for AP (ApcA1-ApcB1; black line) from *Leptolyngbya* sp. JSC-1 and for AP-B (ApcD1-ApcB; red line) and FRL-AP (ApcD4-ApcB3; blue line) from the thermophilic cyanobacterium *Synechococcus* sp. A1463.

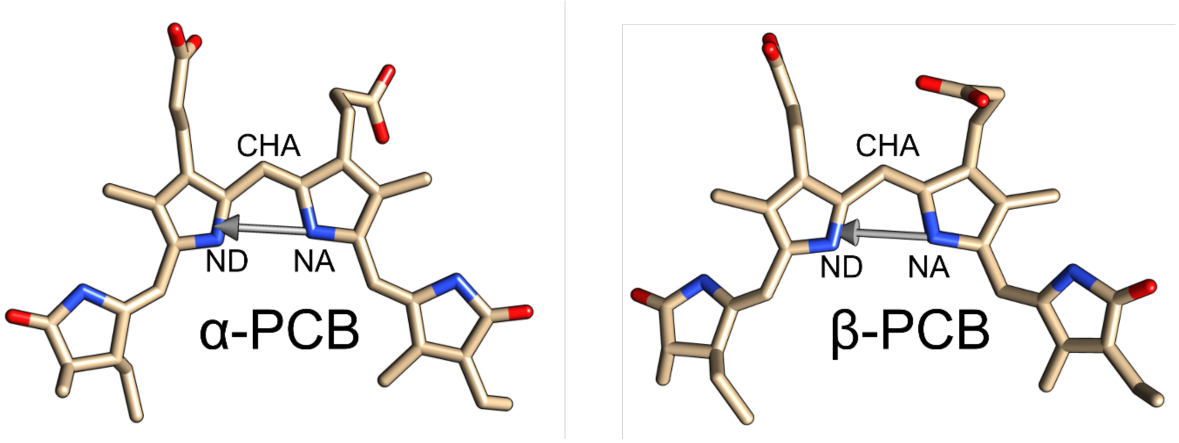

Fig. S11. Calculated orientation of the TDM for the two PCB chromophores found in FRL-AP. The left panel shows the α-PCB and the right panel shows the β-PCB. The grey arrow shows the calculated TDM. The names of the atoms that were used for the FRET calculations based on the FRL-AP cryo-EM structural coordinates are also indicated.

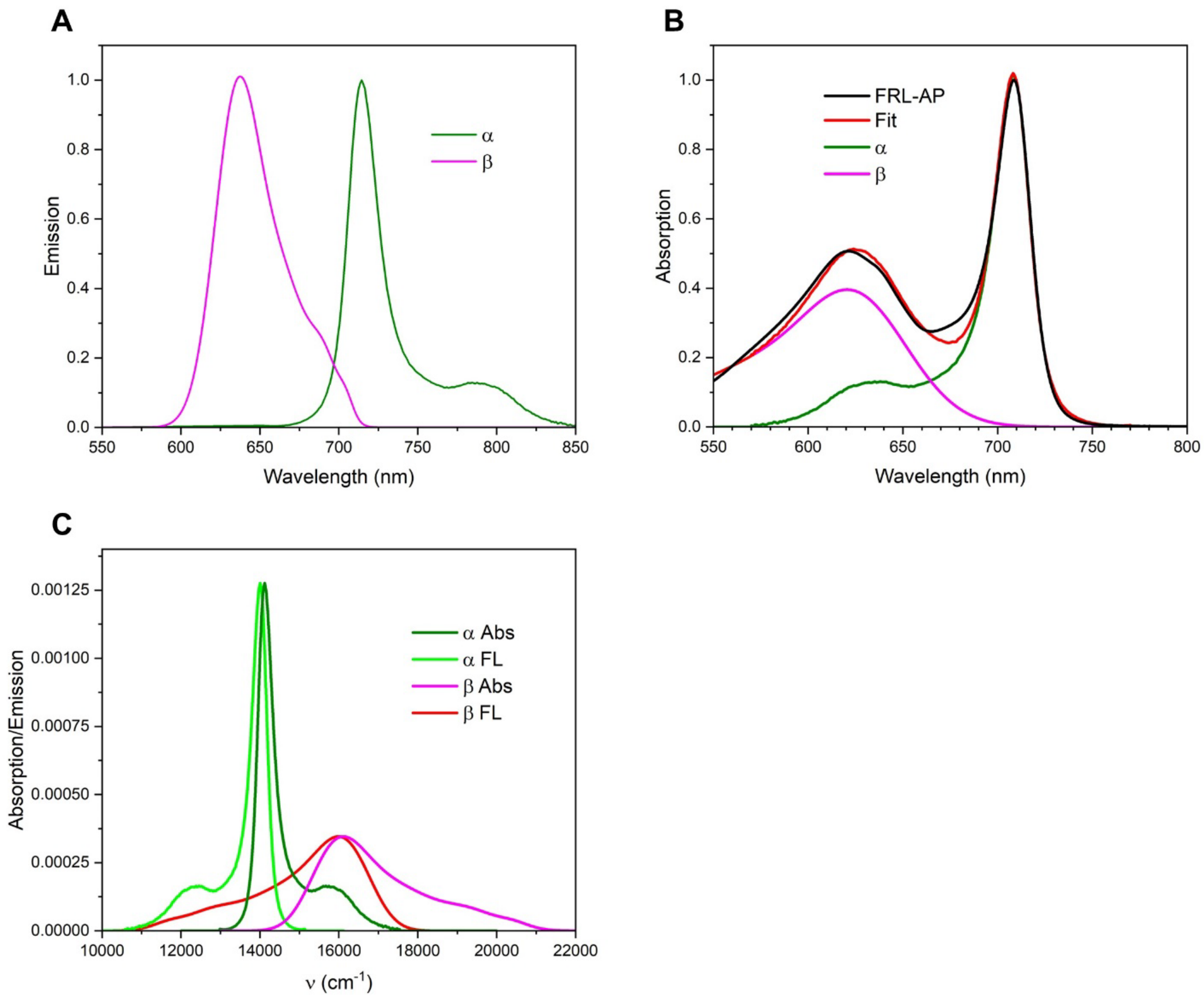

Fig. S12. Steady-state emission and absorption spectra for FRET calculations. (**A**) Emission spectra. The green line shows the room temperature emission spectrum of the α-subunit and the magenta line shows the emission spectrum of the β-subunit. (**B**) Absorption spectrum of FRL-AP (black) and the fitted spectra of the absorption of α-PCB (green), β-PCB (magenta) and of α + β-PCB (red). (**C**) Spectra used for determining the overlap integral value J (see **Materials and Methods**).

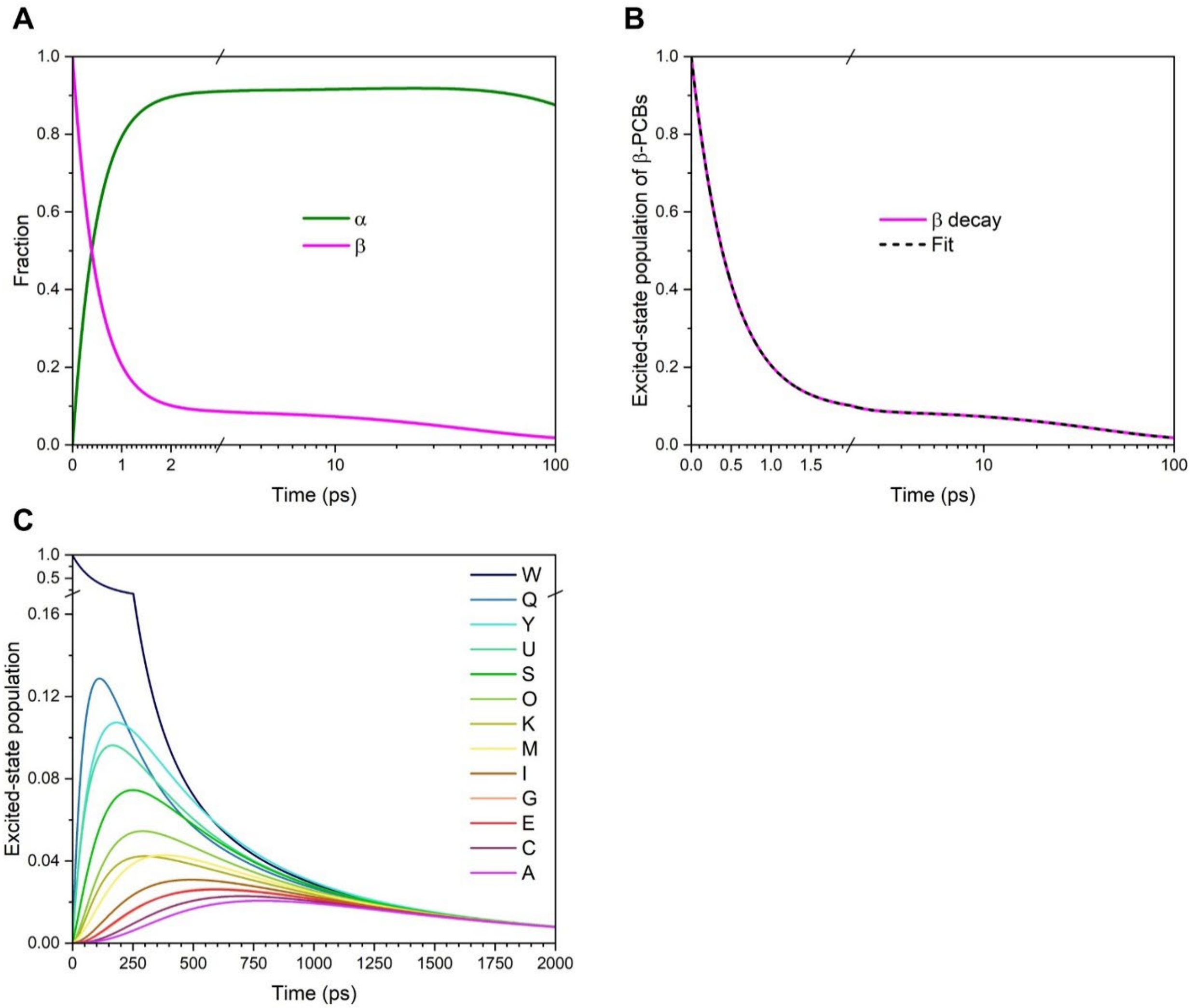

Fig. S13. Modeled EET dynamics. (**A**) Simulated time-evolution of the excited-state populations of the α- and β-PCBs given that all β-PCBs were initially evenly excited. (**B**) The simulated decay of the excited-state populations of the β-PCBs (magenta), given that all β-PCBs were initially evenly excited, and the fitted tri-exponential curve ($0.910e^{-\frac{t}{488\,fs}} + 0.076e^{-\frac{t}{39.7\,ps}} + 0.014e^{-\frac{t}{889\,ps}}$) (black dashed). (**C**) Time-evolution of excited-state populations of α-PCBs after excitation of the terminal α-PCB (W). This was calculated from a model containing 26 PCBs, sequentially labeled A-Z.

A

```
IsiX MNAKKMHPSGYPWWLGNARLMNLSNTFIVAHVAHAALIMAWAGGFTLFELAKFSPERPMY 60
IsiA MQTYNNPEVTYDWWAGNARFANLSGLFIAAHVAQAALIMFWAGAFTLYEISWLTADQSMG 60
     *:: :     * ** ****: ***. **.****:***** ***.***:*:: :: :: *

IsiX EQGLILLPHLATLGWGVGPGGQIVNTFPYVAIASIHLVAAGVLAGGAYFHQTQLPPSLDM 120
IsiA EQGLILLPHLATLGLGVGDGGQVTDTYPLFVVGAVHLIASAVLGAGALFHTFRAPSDLAA 120
     ************** *** ***:.:*:* ..:.::**:*:.**..** **  : * .*

IsiX EFGRAAKFHFTWDDAKTLGVILGHHLLILGLGSLLLVAKAMVFGGLYDANTGQVRLVTAP 180
IsiA ASGAAKRFHFDWNDPKQLGLILGHHLLFLGVGALLLVAKATTWGGLYDAASQTVRLVTEP 180
       * * :*** *:* * **:*******:**:*:******* .:****** :  ***** *

IsiX TLDFATLWNYRTHLFDVNNLEDLVGGHIYVGALLLLGGVWHILVPPFEWVKARFLFSADG 240
IsiA TLNPAVIYGYQTHFASIDNLEDLVGGHVYVGVMLIAGGIWHILVPPFQWTKKVLIYSGEA 240
     **: *.::.*:**: .::*********:***.:*: **:********:*.*  :::*.:.

IsiX ILSYSLFGIALAGFAASYYCGFNSLAYPVEFYGPTLELKSAFTPYYFDPNQTGAWNYSSR 300
IsiA ILSYSLGGIALAGFVAAYFCAVNTLAYPVEFYGAPLEIKLGVTPYFADTVQLPFGAHTPR 300
     ****** *******.*:*:*..*:*********  **:* ..***: *  *     :: *

IsiX VWLANAHFYLAFFFLQGSLWHFQRAMGFDVGKMLQLWQQSRQEMTSQSVYQVQFQCRPQP 360
IsiA AWLSNAHFFLAFFCLQGHLWHALRAMGFDFRRVEKALSSVEA------------------ 342
     .**:****:**** *** ***  ******. :: :  .. .

IsiX DFATFYEPPFAGPEPLSVSPEDIYWYQPPKAPSSRSLSLINGVRQTLYEVNYTLRRWIFY 420
IsiA ------------------------------------------------------------ 342


IsiX EPAREKIAFNRSDLKADYGSRKSRADFSKFARSLPKVFYEPSRVQA 466
IsiA ---------------------------------------------- 342
```

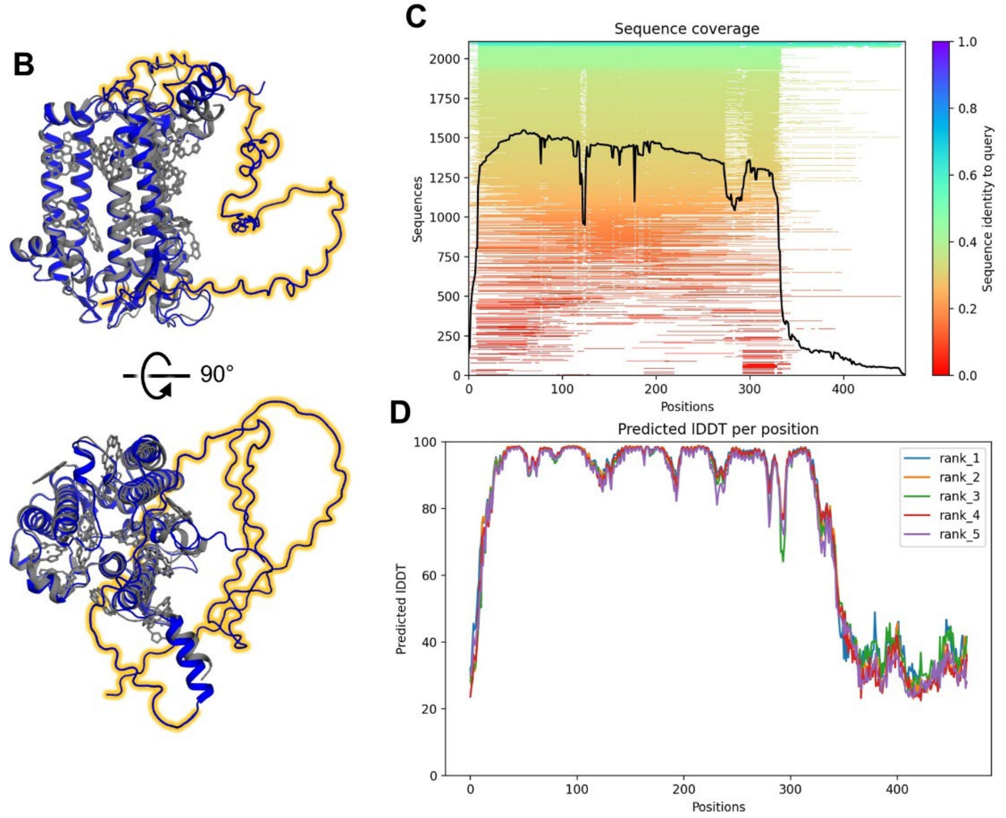

Fig. S14. IsiX comparison with IsiA and structural prediction. (**A**) Residues in IsiA involved in providing axial ligation to Chl molecules (PDB 6KIG, (*25*)) are highlighted in yellow. Clustal

Omega sequence conservation identifiers are shown below the alignment. The C-terminal extension of IsiX is highlighted in orange. (**B**) AlphaFold (*51*) model prediction (blue) superimposed onto the structure if IsiA (PDB 6KIG). The structure corresponding to the C-terminal extension of IsiX is highlighted in orange. (**C**) Sequence coverage output from AlphaFold. (**D**) lDDT score (*52*) output from AlphaFold.

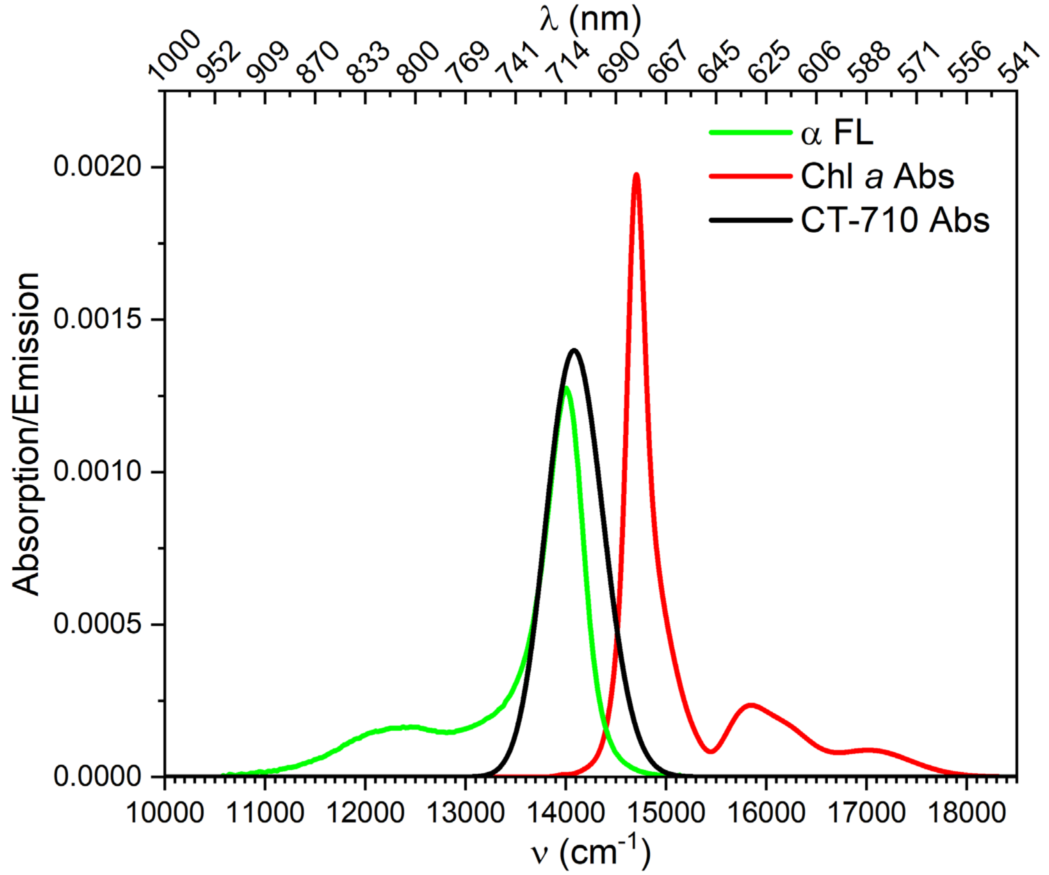

Fig. S15. Absorption spectrum of typical Chl *a* and of a CT-state centered at 710 nm and of the emission spectrum of α-PCBs. The far greater overlap between the emission spectrum of α-PCBs and the absorption spectrum of the 710 nm CT state than between α-PCBs emission and the typical Chl *a* absorption makes it likely that the FRL-AP emission is close to the absorption of PSI red-form Chls.

A

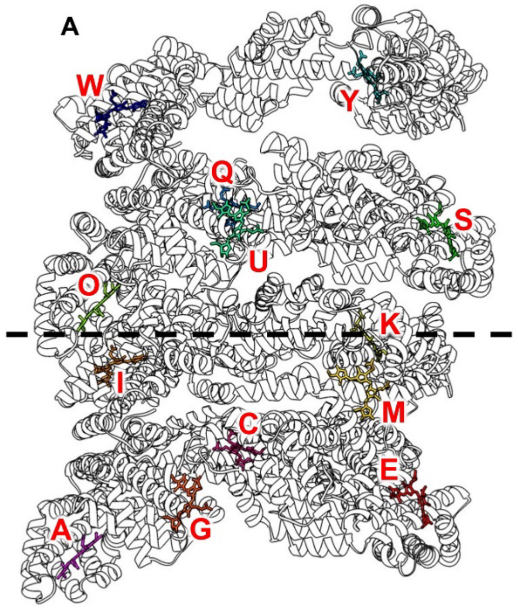

B

Full FRL-AP

	Trap-E	Trap-G	Trap-A
EET efficiency	40%	46%	32%

C

Half FRL-AP

	Trap-S	Trap-U	Trap-O
EET efficiency	65%	65%	57%

Fig. S16. EET efficiency of FRL-AP. (A) Model used for efficiency calculations. Only α-PCBs are shown. **(B)** Average efficiency of energy transfer using a 26-pigment model with traps connected to the α-PCB of E, G, and A. **(C)** Average efficiency of energy transfer using a 13-pigment model with traps connected to the α-PCB of S, U, and O.

Table S1.

Identification of protein subunits the FRL-AP protein purification by tryptic peptide fingerprinting and MS/MS mass spectrometry. Only the top 10 most prevalent proteins are shown. The two most prevalent proteins are shaded in orange.

**Description**	**Coverage (%)**	**# Peptides**
A1463_ApcB3	63	18
A1463_His_ApcD4	49	14
Elongation factor Tu OS=*Synechococcus* sp. (strain ATCC 27264 / PCC 7002 / PR-6)	13	9
Elongation factor tufA (Fragment) OS=*Synechococcus* sp. (strain ATCC 27264 / PCC 7002 / PR-6)	19	7
S-layer like protein probable porin OS=*Synechococcus* sp. (strain ATCC 27264 / PCC 7002 / PR-6)	17	9
1,4-alpha-glucan branching enzyme GlgB OS=*Synechococcus* sp. (strain ATCC 27264 / PCC 7002 / PR-6)	14	9
Glutamine synthetase I beta OS=*Synechococcus* sp. (strain ATCC 27264 / PCC 7002 / PR-6)	8	6
Ribulose bisphosphate carboxylase large chain OS=*Synechococcus* sp. (strain ATCC 27264 / PCC 7002 / PR-6)	9	4
Outer membrane protein, OMP85 family OS=*Synechococcus* sp. (strain ATCC 27264 / PCC 7002 / PR-6)	7	4
Pentapeptide repeats protein OS=*Synechococcus* sp. (strain ATCC 27264 / PCC 7002 / PR-6)	4	1

Table S2.

Cryo-EM data collection, refinement, and validation statistics for helical FRL-AP.

**Data collection and processing**	
Magnification	×105,000
Voltage (kV)	300
Electron exposure (e^{-}Å^{-2})	59.8
Defocus range (μm)	–0.8 to –2.0
Pixel size (Å)	0.413
Symmetry imposed	helical
Initial particle images (no.)	601,552
Final particle images (no.)	74,095
Helical rise (Å)	10.37
Helical twist (°)	101.57
Map resolution (Å)	2.89
FSC threshold	0.143
**Refinement**	
Initial model used (PDB code)	4PO5
Model resolution (Å)	2.9
FSC threshold	0.5
Map resolution range (Å)	2.5 to 3.3
Map-sharpening *B* factor (Å^2)	–93.8
Model composition	
Non-hydrogen atoms	7,968
Protein residues	954
Ligands	9
B factors (Å^2)	
Protein	28.3
Ligands	14.6
R.m.s. deviations	
Bond lengths (Å)	0.011
Bond angles (°)	1.772
**Validation**	
MolProbity	2.19
Clashscore	6.05
Rotamer outliers (%)	4.58
Ramachandran plot	
Favored (%)	94.86
Allowed (%)	5.14
Disallowed (%)	0

REFERENCES AND NOTES

1. R. E. Blankenship, *Molecular Mechanisms of Photosynthesis* (Blackwell Scientific, 2021).

2. M. F. Hohmann-Marriot, R. E. Blankenship, Evolution of photosynthesis. *Annu. Rev. Plant Biol.* **62**, 515–548 (2011).

3. C. J. Gisriel, C. Azai, T. Cardona, Recent advances in the structural diversity of reaction centers. *Photosynth. Res.* **149**, 329–343 (2021).

4. D. A. Bryant, D. P. Canniffe, How nature designs light-harvesting antenna systems: Design principles and functional realization in chlorophototrophic prokaryotes. *J. Phys. B At. Mol. Opt. Phys.* **51**, 033001 (2018).

5. R. Croce, H. van Amerongen, Light harvesting in oxygenic photosynthesis: Structural biology meets spectroscopy. *Science* **369**, eaay2058 (2020).

6. D. A. Bryant, The molecular biology of cyanobacteria, in *Advances in Photosynthesis and Respiration, Vol. 1* (Springer, 1994).

7. W. A. Sidler, Phycobilisome and phycobiliprotein structures, in *Advances in Photosynthesis and Respiration, Vol. 1*, *The Molecular Biology of Cyanobacteria*, Ed. D. A. Bryant (Springer, 1994), pp. 139–216.

8. H. Liu, H. Zhang, D. M. Niedzwiedzki, M. Prado, G. He, M. L. Gross, R. E. Blankenship, Phycobilisomes supply excitations to both photosystems in a megacomplex in cyanobacteria. *Science* **342**, 1104–1107 (2013).

9. D. A. Bryant, G. Guglielmi, N. T. de Marsac, A.-M. Castets, G. Cohen-Bazire, The structure of cyanobacterial phycobilisomes: A model. *Arch. Microbiol.* **123**, 113–127 (1979).

10. L. Zheng, Z. Zheng, X. Li, G. Wang, K. Zhang, P. Wei, J. Zhao, N. Gao, Structural insight into the mechanism of energy transfer in cyanobacterial phycobilisomes. *Nat. Commun.* **12**, 5497 (2021).

11. M. A. Domínguez-Martín, P. V. Sauer, H. Kirst, M. Sutter, D. Bína, B. J. Greber, E. Nogales, T. Polívka, C. A. Kerfeld, Structures of a phycobilisome in light-harvesting and photoprotected states. *Nature* **609**, 835–845 (2022).

12. M.-Y. Ho, N. T. Soulier, D. P. Canniffe, G. Shen, D. A. Bryant, Light regulation of pigment and photosystem biosynthesis in cyanobacteria. *Curr. Opin. Plant Biol.* **37**, 24–33 (2017).

13. F. Wang, M. Chen, Chromatic acclimation processes and their relationships with phycobiliprotein complexes. *Microorganisms* **10**, 1562 (2022)

14. M. Watanabe, D. A. Semchonok, M. T. Webber-Birungi, S. Ehira, K. Kondo, R. Narikawa, M. Ohmori, E. J. Boekema, M. Ikeuchi, Attachment of phycobilisomes in an antenna—Photosystem I supercomplex of cyanobacteria. *Proc. Natl. Acad. Sci. U.S.A.* **111**, 2512–2517 (2014).

15. Y. Li, Y. Lin, C. J. Garvey, D. Birch, R. W. Corkery, P. C. Loughlin, H. Scheer, R. D. Willows, M. Chen, Characterization of red-shifted phycobilisomes isolated from the chlorophyll *f*-containing cyanobacterium *Halomicronema hongdechloris*. *Biochim. Biophys. Acta Bioenerg.* **1857**, 107–114 (2016).

16. M.-Y. Ho, F. Gan, G. Shen, D. A. Bryant, Far-red light photoacclimation (FaRLiP) in *Synechococcus* sp. PCC 7335. II. Characterization of phycobiliproteins produced during acclimation to far-red light. *Photosynth. Res.* **131**, 187–202 (2017).

17. N. Soulier, T. N. Laremore, D. A. Bryant, Characterization of cyanobacterial allophycocyanins absorbing far-red light. *Photosynth. Res.* **145**, 189–207 (2020).

18. N. Soulier, K. Walters, T. N. Laremore, G. Shen, J. H. Golbeck, D. A. Bryant, Acclimation of the photosynthetic apparatus to low light in a thermophilic *Synechococcus* sp. strain. *Photosynth. Res.* **153**, 21–42 (2022).

19. F. Gan, S. Zhang, N. C. Rockwell, S. S. Martin, J. C. Lagarias, D. A. Bryant, Extensive remodeling of a cyanobacterial photosynthetic apparatus in far-red light. *Science* **345**, 1312–1317 (2014).

20. F. Gan, D. A. Bryant, Adaptive and acclimative responses of cyanobacteria to far-red light. *Environ. Microbiol.* **17** 3450–3465 (2015).

21. N. Soulier, D. A. Bryant, The structural basis of far-red light absorbance by allophycocyanins. *Photosynth. Res.* **147**, 11–26 (2021).

22. G. Shen, H. S. Leonard, W. M. Schluchter, D. A. Bryant, CpcM post-translationally methylates asparagine-71/72 of phycobiliprotein beta subunits in *Synechococcus* sp. PCC 7002 and *Synechocystis* sp. PCC 6803. *J. Bacteriol.* **190**, 4808–4817 **(**2008).

23. T. Schirmer, R. Huber, M. Schneider, W. Bode, M. Miller, M. L. Hackert, Crystal structure analysis and refinement at 2.5 Å of hexameric C-phycocyanin from the cyanobacterium *Agmenellum quadruplicatum*: The molecular model and its implications for light-harvesting. *J. Mol. Biol.* **188**, 651–676 (1986).

24. P.-P. Peng, L. L. Dong, Y. F. Sun, X. L. Zeng, W. L. Ding, H. Scheer, X. Yang, K. H. Zhao, The structure of allophycocyanin B from *Synechocystis* PCC 6803 reveals the structural basis for the extreme redshift of the terminal emitter in phycobilisomes. *Acta Crystallogr. Sect. D* **70**, 2558–2569 (2014).

25. P. Cao, D. Cao, L. Si, X. Su, L. Tian, W. Chang, Z. Liu, X. Zhang, M. Li, Structural basis for energy and electron transfer of the photosystem I–IsiA–Flavodoxin supercomplex. *Nat. Plants* **6**, 167–176 (2020).

26. N. V. Karapetyan, Y. V. Bolychevtseva, N. P. Yurina, I. V. Terekhova, V. V. Shubin, M. Brecht, Long-wavelength chlorophylls in photosystem I of cyanobacteria: Origin, localization, and functions. *Biochemistry (Moscow)* **79**, 213–220 (2014).

27. V. Mascoli, L. Bersanini, R. Croce, Far-red absorption and light-use efficiency trade-offs in chlorophyll *f* photosynthesis. *Nat. Plants* **6**, 1044–1053 (2020).

28. J. Zivanov, T. Nakane, B. O. Forsberg, D. Kimanius, W. J. H. Hagen, E. Lindahl, S. H. W. Scheres, New tools for automated high-resolution cryo-EM structure determination in RELION-3. *eLife* **7**, e42166 (2018).

29. S. Q. Zheng, E. Palovcak, J. P. Armache, K. A. Verba, Y. Cheng, D. A. Agard, MotionCor2: Anisotropic correction of beam-induced motion for improved cryo-electron microscopy. *Nat. Methods* **14**, 331–332 (2017).

30. A. Rohou, N. Grigorieff, CTFFIND4: Fast and accurate defocus estimation from electron micrographs. *J. Struct. Biol.* **192**, 216–221 (2015).

31. P. Emsley, B. Lohkamp, W. G. Scott, K. Cowtan, Features and development of Coot. *Acta Crystallogr. Sect. D Biol. Crystallogr.* **66**, 486–501 (2010).

32. E. F. Pettersen, T. D. Goddard, C. C. Huang, G. S. Couch, D. M. Greenblatt, E. C. Meng, T. E. Ferrin, UCSF Chimera—A visualization system for exploratory research and analysis. *J. Comput. Chem.* **25**, 1605–1612 (2004).

33. N. Guex, M. C. Peitsch, T. Schwede, Automated comparative protein structure modeling with SWISS-MODEL and Swiss-PdbViewer: A historical perspective. *Electrophoresis* **30**, S162–S173 (2009).

34. P. D. Adams, P. V. Afonine, G. Bunkóczi, V. B. Chen, I. W. Davis, N. Echols, J. J. Headd, L. W. Hung, G. J. Kapral, R. W. Grosse-Kunstleve, A. J. McCoy, N. W. Moriarty, R. Oeffner, R. J. Read, D. C. Richardson, J. S. Richardson, T. C. Terwilliger, P. H. Zwart, PHENIX: A comprehensive python-based system for macromolecular structure solution. *Acta Crystallogr. Sect. D Biol. Crystallogr.* **66**, 213–221 (2010).

35. P. V. Afonine, B. K. Poon, R. J. Read, O. V. Sobolev, T. C. Terwilliger, A. Urzhumtsev, P. D. Adams, Real-space refinement in PHENIX for cryo-EM and crystallography. *Acta Crystallogr. Sect. D* **74**, 531–544 (2018).

36. J. J. Snellenburg, S. Laptenok, R. Seger, K. M. Mullen, I. H. M. van Stokkum, Glotaran: A Java-based graphical user interface for the R package TIMP. *J. Stat. Softw.* **49**, 1–22 (2012).

37. A. V. Digris, E. G. Novikov, V. V. Skakun, V. V. Apanasovich, "Global a nalysis of t ime-r esolved f luorescence data," in *Fluorescence Spectroscopy and Microscopy: Methods and Protocols*, Y. Engelborghs, A. J. W. G. Visser, Eds. (Humana Press, 2014), pp. 257–277.

38. S. J. A. van Gisbergen, J. G. Snijders, E. J. Baerends, Implementation of time-dependent density functional response equations. *Comput. Phys. Commun.* **118**, 119–138 (1999).

39. G. te Velde, F. M. Bickelhaupt, E. J. Baerends, C. Fonseca Guerra, S. J. A. van Gisbergen, J. G. Snijders, T. Ziegler, Chemistry with ADF. *J. Comput. Chem.* **22**, 931–967 (2001).

40. A. B. Doust, C. N. J. Marai, S. J. Harrop, K. E. Wilk, P. M. G. Curmi, G. D. Scholes, Developing a structure–function model for the cryptophyte phycoerythrin 545 using ultrahigh resolution crystallography and ultrafast laser spectroscopy. *J. Mol. Biol.* **344**, 135–153 (2004).

41. R. R. Sonani, G. D. Gupta, D. Madamwar, V. Kumar, Crystal structure of allophycocyanin from marine cyanobacterium *Phormidium* sp. A09DM. *PLOS ONE* **10**, e0124580 (2015).

42. J. M. Womick, A. M. Moran, Exciton coherence and energy transport in the light-harvesting dimers of allophycocyanin. *J. Phys. Chem. B* **113**, 15747–15759 (2009).

43. A. D. Becke, Density-functional thermochemistry. III. The role of exact exchange. *J. Chem. Phys.* **98**, 5648–5652 (1993).

44. C. Lee, W. Yang, R. G. Parr, Development of the Colle-Salvetti correlation-energy formula into a functional of the electron density. *Phys. Rev. B* **37**, 785–789 (1988).

45. P. J. Stephens, F. J. Devlin, C. F. Chabalowski, M. J. Frisch, Ab initio calculation of vibrational absorption and circular dichroism spectra using density functional force fields. *J. Phys. Chem.* **98**, 11623–11627 (1994).

46. C. Curutchet, V. I. Novoderezhkin, J. Kongsted, A. Muñoz-Losa, R. van Grondelle, G. D. Scholes, B. Mennucci, Energy flow in the cryptophyte PE545 antenna is directed by bilin pigment conformation. *J. Phys. Chem. B* **117**, 4263–4273 (2013).

47. Y. Ren, B. Chi, O. Melhem, K. Wei, L. Feng, Y. Li, X. Han, D. Li, Y. Zhang, J. Wan, X. Xu, M. Yang, Understanding the electronic energy transfer pathways in the trimeric and hexameric aggregation state of cyanobacteria phycocyanin within the framework of Förster theory. *J. Comput. Chem.* **34**, 1005–1012 (2013).

48. G. Cinque, R. Croce, R. Bassi, Absorption spectra of chlorophyll *a* and *b* in Lhcb protein environment. *Photosynth. Res.* **64**, 233–242 (2000).

49. V. I. Novoderezhkin, R. Croce, M. Wahadoszamen, I. Polukhina, E. Romero, R. van Grondelle, Mixing of exciton and charge-transfer states in light-harvesting complex Lhca4. *Phys. Chem. Chem. Phys.* **18**, 19368–19377 (2016).

50. F. Sievers, A. Wilm, D. Dineen, T. J. Gibson, K. Karplus, W. Li, R. Lopez, H. M. William, M. Remmert, J. Söding, J. D. Thompson, D. G. Higgins, Fast, scalable generation of high quality protein multiple sequence alignments using Clustal Omega. *Mol. Syst. Biol.* **7**, 1–6 (2011).

51. J. Jumper, R. Evans, A. Pritzel, T. Green, M. Figurnov, O. Ronneberger, K. Tunyasuvunakool, R. Bates, A. Žídek, A. Potapenko, A. Bridgland, C. Meyer, S. A. A. Kohl, A. J. Ballard, A. Cowie, B. Romera-Paredes, S. Nikolov, R. Jain, J. Adler, T. Back, S. Petersen, D. Reiman, E. Clancy, M. Zielinski, M. Steinegger, M. Pacholska, T. Berghammer, S. Bodenstein, D. Silver, O. Vinyals, A. W. Senior, K. Kavukcuoglu, P. Kohli, D. Hassabis, Highly accurate protein structure prediction with AlphaFold. *Nature* **596**, 583–589 (2021).

52. V. Mariani, M. Biasini, A. Barbato, T. Schwede, lDDT: A local superposition-free score for comparing protein structures and models using distance difference tests. *Bioinformatics* **29**, 2722–2728 (2013).
